# Supplementary material for: VolcanoSV enables accurate and robust structural variant calling in diploid genomes from single-molecule long read sequencing
Source: Nat Commun. 2024 Aug 13;15:6956. doi: 10.1038/s41467-024-51282-0 (PMC11322167; doi:10.1038/s41467-024-51282-0)
Supplement: Supplementary file 1 — Supplementary Information [file 41467_2024_51282_MOESM1_ESM.pdf]

VolcanoSV enables accurate and robust structural variant calling in diploid genomes from single-molecule long read sequencing

Can Luo, Yichen Henry Liu, and Xin Maizie Zhou

Contents

1 Supplementary Notes **2**
1.1 Analyzing the low recall of complex SVs in real cancer datasets 2

2 Supplementary Tables **3**
1 SV evaluation against GIAB benchmark on five Hifi datasets by VolcanoSV 3
2 SV evaluation against GIAB benchmark on three CLR datasets by VolcanoSV . . . 4
3 SV evaluation against GIAB benchmark on six ONT datasets by VolcanoSV 5
4 SV annotation using Ensembl Variant Effect Predictor (VEP) and Genome Aggregation Database (gnomAD) for all datasets. 6
5 SV annotation using Ensembl Variant Effect Predictor (VEP) and Genome Aggregation Database (gnomAD) for three representative datasets. 7
6 Subsampling effects on Hifi_L1 8
7 Subsampling effects on CLR_L1 9
8 Subsampling effects on ONT_L1 10
9 SV evaluation against GIAB benchmark on five Hifi datasets by VolcanoSV-vc . . . 11
10 SV evaluation against GIAB benchmark on three CLR datasets by VolcanoSV-vc . . 12
11 SV evaluation against GIAB benchmark on six ONT datasets by VolcanoSV-vc . . . 13
12 SNP and small indel evaluation against GIAB benchmark on five Hifi datasets . . . 14
13 SNP and small indel evaluation against GIAB benchmark on three CLR datasets . . 15
14 SNP and small indel evaluation against GIAB benchmark on six ONT datasets . . . 16
15 Computing resource consumption of assemblers 17
16 Computing resource consumption of SV callers 17

3 Supplementary Figures **18**
1 The consequences annotation of unique TP SVs with correct GT detected by VolcanoSV for all 14 libraries using Ensembl Variant Effect Predictor (VEP) 19
2 The consequences annotation of unique TP SVs detected by VolcanoSV for three representative libraries using Ensembl Variant Effect Predictor (VEP) 20
3 F1 accuracy of SV detection at different size ranges 21
4 Analyzing the low recall of complex SVs in real cancer datasets 22
5 F1 accuracy by tuning different evaluation parameters and distribution of breakpoint shift and alternate allele sequence similarity for SVs on CLR_L1 23
6 F1 accuracy by tuning different evaluation parameters and distribution of breakpoint shift and alternate allele sequence similarity for SVs on ONT_L1 24
7 Cross datasets evaluation against GIAB HG002 benchmark by VolcanoSV-vc. 25
8 False discovery analysis in Hifi_L6 on the T2T-CHM13 reference. 26
9 False discovery analysis in Hifi_L7 on the T2T-CHM13 reference. 27

Supplementary References **28**

1 Supplementary Notes

1.1 Analyzing the low recall of complex SVs in real cancer datasets

To investigate the reason for the low recall or whether VolcanoSV is more likely to detect common complex SVs which can be identified by other long-read datasets, we analyzed the overlapping VolcanoSV calls between paired normal and tumor HCC1395 samples, and HG002, using PacBio CLR libraries.

In Supplementary Figure 4a, $HCC1395^N$ denotes the number of SV calls by VolcanoSV for the HCC1395 normal library, $HG002^N$ for the HG002 library, and overlapN for the overlapping SV calls between these two call sets. In Supplementary Figure 4b, $HCC1395^T$ denotes the SV calls by VolcanoSV for the HCC1395 tumor library, $HG002^N$ for the HG002 library, $overlap^T$ for the overlapping SV calls between two sets. For an effective tool, we anticipated similar overlap values ($overlap^N \approx overlap^T$); however, there were significantly more calls for HCC1395 tumor library compared to the HCC1395 normal library. To better illustrate this, we formulated three equations (1-3) shown in Supplementary Figure 4c. To achieve high recall with a tool, we expected the following: Equation 1: $overlap^N$ / $overlap^T \approx 1$, indicating the tool called similar number of germline SVs in both normal and tumor samples, part of which could be common SVs shared with HG002; Equation 2: $overlap^N$ / $HCC1395^N$ – $overlap^T$ / $HCC1395^T > 0$, suggesting the tool called a significant number of somatic SVs in addition to the germline SVs in tumor sample; Equation 3: $overlap^N$ / $HG002^N$ – $overlap^T$ / $HG002^N \approx 0$, showing consistent overlap ratios across HG002 samples (similar as Equation 1). Our results showed that the TRA recall (0.32) and INV recall (0.29) by VolcanoSV outperformed the DUP recall (0.13). This aligns with the expected outcomes derived from the three equations for each SV type as shown in Supplementary Figure 4d. Equation 2, which was -0.16% for DUP, suggested that VolcanoSV detected less SVs in tumor samples compared to normal samples (2379 DUPs in tumor versus 2768 DUPs in normal), implying the low recall for DUP.

To create a gold standard complex SV call set, the original paper by Talsania et al. [1] employed various sequencing technologies for both short and long reads, alongside different alignment-based tools to identify SVs. Specifically, for long-read PacBio CLR datasets, they utilized pbsv and Sniffles. We thus expanded our analysis to incorporate pbsv calls, and pbsv did achieve better recall rates for TRA (0.55), INV (0.48), and DUP (0.43) compared to VolcanoSV. This inclusion could help us gain a more comprehensive understanding of the recall dynamics. The metric values derived from the three equations for pbsv supported previous findings, suggesting that these equations can help explain the varying recall levels observed across different tools. Specifically, equation 2 values, which were 14.41%, 14.14%, and 1.36% for TRA, INV, and DUP, respectively, suggested that pbsv detected more unique complex SVs in tumor samples compared to normal samples to achieve better recall (also indicated by metric values from Equation 1 and 3). However, two important points to consider were: 1) since the high-confidence benchmark callset included SV calls from all alignment-based tools like pbsv, using it to evaluate VolcanoSV might introduce bias; 2) Benchmarking complex SVs is akin to evaluating large indel SVs but may be even more intricate. Evaluation parameters in terms of sequence similarity and breakpoints would greatly impact the recall of a specific tool.

In conclusion, the metric values derived from the three equations partially offer an explanation for low recall rates in complex SVs by VolcanoSV. To improve recall, it is crucial to identify more unique calls in tumor samples that distinctly differ from those in normal samples. However, it is challenging to solely use assembled contigs to detect complex SVs. This could be due to the limitations of assembly algorithms and the complexity of graph construction. Many genome assembly algorithms are designed to create contigs by tracing the simplest paths through a graph of overlapping reads, such as an overlap graph, constructed from long reads. However, these algorithms typically prioritize the most common paths, which can overlook complex structural variants like translocations. Translocations rearrange DNA by moving segments from one part of the genome to another, creating non-linear or non-sequential patterns. These patterns disrupt the graph's continuity and form connections that are challenging to interpret correctly because they deviate from the usual linear arrangement of chromosomes, complicating the detection of such variants.

2 Supplementary Tables

INS		VolcanoSV (2023)	PAV (2021)	SVIM-asm (2020)	Dipcall (2018)
Total Benchmark Calls (>50): 5,281					
	**comp cnt**	4,156	4,158	4,215	4,220
	**TP-comp**	4,964	4,917	4,916	4,824
	**FP**	591	778	766	1,801
	**FN**	317	364	365	457
	**precision**	89.36%	86.34%	86.52%	72.82%
Hifi_L1	**recall**	94.0%	93.11%	93.09%	91.35%
	**F1**	91.62%	89.6%	89.68%	81.03%
	**base cnt**	5,281	5,281	5,281	5,281
	**TP-comp_TP-gt**	4,883	4,699	4,809	3,755
	**TP-comp_FP-gt**	81	218	107	1,069
	**gt_concordance**	98.37%	95.57%	97.82%	77.84%
	**comp cnt**	4,180	4,209	4,230	4,237
	**TP-comp**	4,945	4,929	4,907	4,837
	**FP**	668	779	774	1,776
	**FN**	336	352	374	444
	**precision**	88.1%	86.35%	86.38%	73.14%
Hifi_L2	**recall**	93.64%	93.33%	92.92%	91.59%
	**F1**	90.78%	89.71%	89.53%	81.34%
	**base cnt**	5,281	5,281	5,281	5,281
	**TP-comp_TP-gt**	4,847	4,737	4,793	3,798
	**TP-comp_FP-gt**	98	192	114	1,039
	**gt_concordance**	98.02%	96.1%	97.68%	78.52%
	**comp cnt**	4,182	4,196	4,239	4,245
	**TP-comp**	4,957	4,912	4,919	4,846
	**FP**	678	781	777	1,795
	**FN**	324	369	362	435
	**precision**	87.97%	86.28%	86.36%	72.97%
Hifi_L3	**recall**	93.86%	93.01%	93.15%	91.76%
	**F1**	90.82%	89.52%	89.62%	81.3%
	**base cnt**	5,281	5,281	5,281	5,281
	**TP-comp_TP-gt**	4,877	4,661	4,818	3,794
	**TP-comp_FP-gt**	80	251	101	1,052
	**gt_concordance**	98.39%	94.89%	97.95%	78.29%
	**comp cnt**	4,182	4,175	4,218	4,228
	**TP-comp**	4,958	4,911	4,921	4,832
	**FP**	658	788	769	1,795
	**FN**	323	370	360	449
	**precision**	88.28%	86.17%	86.49%	72.91%
Hifi_L4	**recall**	93.88%	92.99%	93.18%	91.5%
	**F1**	91.0%	89.45%	89.71%	81.16%
	**base cnt**	5,281	5,281	5,281	5,281
	**TP-comp_TP-gt**	4,879	4,681	4,804	3,769
	**TP-comp_FP-gt**	79	230	117	1,063
	**gt_concordance**	98.41%	95.32%	97.62%	78.0%
	**comp cnt**	4,166	4,230	4,233	4,225
	**TP-comp**	4,956	4,956	4,916	4,821
	**FP**	662	778	762	1,782
	**FN**	325	325	365	460
	**precision**	88.22%	86.43%	86.58%	73.01%
Hifi_L5	**recall**	93.85%	93.85%	93.09%	91.29%
	**F1**	90.94%	89.99%	89.72%	81.13%
	**base cnt**	5,281	5,281	5,281	5,281
	**TP-comp_TP-gt**	4,876	4,807	4,797	3,768
	**TP-comp_FP-gt**	80	149	119	1,053
	**gt_concordance**	98.39%	96.99%	97.58%	78.16%

DEL		VolcanoSV (2023)	PAV (2021)	SVIM-asm (2020)	Dipcall (2018)
Total Benchmark Calls (>50): 4,116					
	**comp cnt**	5,555	5,695	5,682	6,625
	**TP-comp**	3,895	3,837	3,865	3,812
	**FP**	261	321	350	408
	**FN**	221	279	251	304
	**precision**	93.72%	92.28%	91.7%	90.33%
Hifi_L1	**recall**	94.63%	93.22%	93.9%	92.61%
	**F1**	94.17%	92.75%	92.79%	91.46%
	**base cnt**	4,116	4,116	4,116	4,116
	**TP-comp_TP-gt**	3,859	3,726	3,782	3,688
	**TP-comp_FP-gt**	36	111	83	124
	**gt_concordance**	99.08%	97.11%	97.85%	96.75%
	**comp cnt**	5,613	5,708	5,681	6,613
	**TP-comp**	3,897	3,874	3,892	3,818
	**FP**	283	335	338	419
	**FN**	219	242	224	298
	**precision**	93.23%	92.04%	92.01%	90.11%
Hifi_L2	**recall**	94.68%	94.12%	94.56%	92.76%
	**F1**	93.95%	93.07%	93.27%	91.42%
	**base cnt**	4,116	4,116	4,116	4,116
	**TP-comp_TP-gt**	3,854	3,768	3,838	3,698
	**TP-comp_FP-gt**	43	106	54	120
	**gt_concordance**	98.9%	97.26%	98.61%	96.86%
	**comp cnt**	5,635	5,693	5,696	6,641
	**TP-comp**	3,910	3,848	3,887	3,818
	**FP**	272	348	352	427
	**FN**	206	268	229	298
	**precision**	93.5%	91.71%	91.7%	89.94%
Hifi_L3	**recall**	95.0%	93.49%	94.44%	92.76%
	**F1**	94.24%	92.59%	93.05%	91.33%
	**base cnt**	4,116	4,116	4,116	4,116
	**TP-comp_TP-gt**	3,871	3,718	3,832	3,697
	**TP-comp_FP-gt**	39	130	55	121
	**gt_concordance**	99.0%	96.62%	98.59%	96.83%
	**comp cnt**	5,616	5,699	5,690	6,627
	**TP-comp**	3,913	3,845	3,886	3,811
	**FP**	269	330	332	417
	**FN**	203	271	230	305
	**precision**	93.57%	92.1%	92.13%	90.14%
Hifi_L4	**recall**	95.07%	93.42%	94.41%	92.59%
	**F1**	94.31%	92.75%	93.26%	91.35%
	**base cnt**	4,116	4,116	4,116	4,116
	**TP-comp_TP-gt**	3,870	3,726	3,835	3,684
	**TP-comp_FP-gt**	43	119	51	127
	**gt_concordance**	98.9%	96.91%	98.69%	96.67%
	**comp cnt**	5,618	5,734	5,678	6,603
	**TP-comp**	3,905	3,891	3,891	3,804
	**FP**	261	339	342	421
	**FN**	211	225	225	312
	**precision**	93.73%	91.99%	91.92%	90.04%
Hifi_L5	**recall**	94.87%	94.53%	94.53%	92.42%
	**F1**	94.3%	93.24%	93.21%	91.21%
	**base cnt**	4,116	4,116	4,116	4,116
	**TP-comp_TP-gt**	3,872	3,838	3,835	3,686
	**TP-comp_FP-gt**	33	53	56	118
	**gt_concordance**	99.15%	98.64%	98.56%	96.9%

Supplementary Table 1: Genome-wide SV (≥50bp) evaluation against GIAB benchmark on **five Hifi** datasets (p=0.5, P=0.5, r=500, and O=0.01 for Truvari). The left and right panels are for insertions (INS) and deletions (DEL), respectively. The evaluation metrics are true positive (TP-comp), false positive (FP), false negative (FN), precision, recall, F1, true positive for genotype accuracy (TP-comp_TP-gt), false positive for genotype accuracy (TP-comp_FP-gt), and genotype accuracy (gt_concordance). For TP-comp, precision, recall, F1, TP-comp_TP-gt, and gt_concordance, the highest values across all tools are highlighted in green color. The evaluated assembly-based tools are **VolcanoSV**, PAV, SVIM-asm, and Dipcall. Source data are provided as a Source Data file.

INS		VolcanoSV (2023)	PAV (2021)	SVIM-asm (2020)	Dipcall (2018)
Total Benchmark Calls (>50): 5,281					
CLR_L1	**comp cnt**	4,125	4,017	4,103	4,030
	**TP-comp**	4,943	4,846	4,928	4,654
	**FP**	762	913	896	2,317
	**FN**	338	435	353	627
	**precision**	86.64%	84.15%	84.62%	66.76%
	**recall**	93.6%	91.76%	93.32%	88.13%
	**F1**	89.99%	87.79%	88.75%	75.97%
	**base cnt**	5,281	5,281	5,281	5,281
	**TP-comp_TP-gt**	4,863	4,646	4,769	3,146
	**TP-comp_FP-gt**	80	200	159	1,508
	**gt_concordance**	98.38%	95.87%	96.77%	67.6%
CLR_L2	**comp cnt**	4,154	4,387	4,004	1,051
	**TP-comp**	4,882	4,815	4,750	1,097
	**FP**	773	1,868	1,172	479
	**FN**	399	466	531	4,184
	**precision**	86.33%	72.05%	80.21%	69.61%
	**recall**	92.44%	91.18%	89.95%	20.77%
	**F1**	89.28%	80.49%	84.8%	32.0%
	**base cnt**	5,281	5,281	5,281	5,281
	**TP-comp_TP-gt**	4,691	4,418	4,370	746
	**TP-comp_FP-gt**	191	397	380	351
	**gt_concordance**	96.09%	91.75%	92.0%	68.0%
CLR_L3	**comp cnt**	4,105	3,931	3,829	2,296
	**TP-comp**	4,899	4,843	4,774	2,497
	**FP**	720	1,294	1,083	1,637
	**FN**	382	438	507	2,784
	**precision**	87.19%	78.91%	81.51%	60.4%
	**recall**	92.77%	91.71%	90.4%	47.28%
	**F1**	89.89%	84.83%	85.72%	53.04%
	**base cnt**	5,281	5,281	5,281	5,281
	**TP-comp_TP-gt**	4,740	4,322	4,290	1,355
	**TP-comp_FP-gt**	159	521	484	1,142
	**gt_concordance**	96.75%	89.24%	89.86%	54.27%

DEL		VolcanoSV (2023)	PAV (2021)	SVIM-asm (2020)	Dipcall (2018)
Total Benchmark Calls (>50): 4,116					
CLR_L1	**comp cnt**	5,705	5,759	5,824	6,971
	**TP-comp**	3,880	3,664	3,775	3,579
	**FP**	245	353	328	451
	**FN**	236	452	341	537
	**precision**	94.06%	91.21%	92.01%	88.81%
	**recall**	94.27%	89.02%	91.72%	86.95%
	**F1**	94.16%	90.1%	91.86%	87.87%
	**base cnt**	4,116	4,116	4,116	4,116
	**TP-comp_TP-gt**	3,841	3,605	3,717	3,377
	**TP-comp_FP-gt**	39	59	58	202
	**gt_concordance**	98.99%	98.39%	98.46%	94.36%
CLR_L2	**comp cnt**	5,655	6,683	5,922	1,576
	**TP-comp**	3,866	3,576	3,544	866
	**FP**	288	811	460	185
	**FN**	250	540	572	3,250
	**precision**	93.07%	81.51%	88.51%	82.4%
	**recall**	93.93%	86.88%	86.1%	21.04%
	**F1**	93.49%	84.11%	87.29%	33.52%
	**base cnt**	4,116	4,116	4,116	4,116
	**TP-comp_TP-gt**	3,816	3,433	3,375	740
	**TP-comp_FP-gt**	50	143	169	126
	**gt_concordance**	98.71%	96.0%	95.23%	85.45%
CLR_L3	**comp cnt**	5,619	6,137	5,857	4,134
	**TP-comp**	3,841	3,472	3,469	1,907
	**FP**	264	459	360	389
	**FN**	275	644	647	2,209
	**precision**	93.57%	88.32%	90.6%	83.06%
	**recall**	93.32%	84.35%	84.28%	46.33%
	**F1**	93.44%	86.29%	87.33%	59.48%
	**base cnt**	4,116	4,116	4,116	4,116
	**TP-comp_TP-gt**	3,766	3,321	3,336	1,607
	**TP-comp_FP-gt**	75	151	133	300
	**gt_concordance**	98.05%	95.65%	96.17%	84.27%

Supplementary Table 2: Genome-wide SV (≥50bp) evaluation against GIAB benchmark on **three CLR** datasets (p=0.5, P=0.5, r=500, and O=0.01 for Truvari). The left and right panels are for insertions (INS) and deletions (DEL), respectively. The evaluation metrics are true positive (TP-comp), false positive (FP), false negative (FN), precision, recall, F1, true positive for genotype accuracy (TP-comp_TP-gt), false positive for genotype accuracy (TP-comp_FP-gt), and genotype accuracy (gt_concordance). For TP-comp, precision, recall, F1, TP-comp_TP-gt, and gt_concordance, the highest values across all tools are highlighted in green color. The evaluated assembly-based tools are **VolcanoSV**, PAV, SVIM-asm, and Dipcall. Source data are provided as a Source Data file.

INS		VolcanoSV (2023)	PAV (2021)	SVIM-asm (2020)	Dipcall (2018)
Total Benchmark Calls (>50): 5,281					
ONT_L1	**comp cnt**	4,156	4,113	4,238	4,338
	**TP-comp**	4,936	4,828	4,950	4,770
	**FP**	695	832	835	2,514
	**FN**	345	453	331	511
	**precision**	87.66%	85.3%	85.57%	65.49%
	**recall**	93.47%	91.42%	93.73%	90.32%
	**F1**	90.47%	88.26%	89.46%	75.93%
	**base cnt**	5,281	5,281	5,281	5,281
	**TP-comp_TP-gt**	4,858	4,699	4,855	3,039
	**TP-comp_FP-gt**	78	129	95	1,731
	**gt_concordance**	98.42%	97.33%	98.08%	63.71%
ONT_L2	**comp cnt**	4,156	4,296	4,288	4,369
	**TP-comp**	4,855	4,909	4,887	4,478
	**FP**	638	884	891	2,489
	**FN**	426	372	394	803
	**precision**	88.39%	84.74%	84.58%	64.27%
	**recall**	91.93%	92.96%	92.54%	84.79%
	**F1**	90.12%	88.66%	88.38%	73.12%
	**base cnt**	5,281	5,281	5,281	5,281
	**TP-comp_TP-gt**	4,742	4,773	4,781	2,671
	**TP-comp_FP-gt**	113	136	106	1,807
	**gt_concordance**	97.67%	97.23%	97.83%	59.65%
ONT_L3	**comp cnt**	4,166	4,192	4,254	4,318
	**TP-comp**	4,938	4,896	4,929	4,724
	**FP**	717	795	851	2,492
	**FN**	343	385	352	557
	**precision**	87.32%	86.03%	85.28%	65.47%
	**recall**	93.51%	92.71%	93.33%	89.45%
	**F1**	90.31%	89.25%	89.12%	75.6%
	**base cnt**	5,281	5,281	5,281	5,281
	**TP-comp_TP-gt**	4,850	4,760	4,840	3,011
	**TP-comp_FP-gt**	88	136	89	1,713
	**gt_concordance**	98.22%	97.22%	98.19%	63.74%
ONT_L4	**comp cnt**	4,131	4,223	4,203	3,780
	**TP-comp**	4,878	4,840	4,811	3,881
	**FP**	802	1,006	995	2,324
	**FN**	403	441	470	1,400
	**precision**	85.88%	82.79%	82.86%	62.55%
	**recall**	92.37%	91.65%	91.1%	73.49%
	**F1**	89.01%	87.0%	86.79%	67.58%
	**base cnt**	5,281	5,281	5,281	5,281
	**TP-comp_TP-gt**	4,747	4,637	4,646	2,152
	**TP-comp_FP-gt**	131	203	165	1,729
	**gt_concordance**	97.31%	95.81%	96.57%	55.45%
ONT_L5	**comp cnt**	4,150	4,033	4,236	4,308
	**TP-comp**	4,918	4,713	4,906	4,584
	**FP**	678	850	862	2,598
	**FN**	363	568	375	697
	**precision**	87.88%	84.72%	85.06%	63.83%
	**recall**	93.13%	89.24%	92.9%	86.8%
	**F1**	90.43%	86.92%	88.8%	73.56%
	**base cnt**	5,281	5,281	5,281	5,281
	**TP-comp_TP-gt**	4,816	4,580	4,804	2,716
	**TP-comp_FP-gt**	102	133	102	1,868
	**gt_concordance**	97.93%	97.18%	97.92%	59.25%
ONT_L6	**comp cnt**	4,135	4,113	4,237	4,297
	**TP-comp**	4,893	4,791	4,894	4,594
	**FP**	669	860	899	2,537
	**FN**	388	490	387	687
	**precision**	87.97%	84.78%	84.48%	64.42%
	**recall**	92.65%	90.72%	92.67%	86.99%
	**F1**	90.25%	87.65%	88.39%	74.03%
	**base cnt**	5,281	5,281	5,281	5,281
	**TP-comp_TP-gt**	4,816	4,652	4,801	2,783
	**TP-comp_FP-gt**	77	139	93	1,811
	**gt_concordance**	98.43%	97.1%	98.1%	60.58%

DEL		VolcanoSV (2023)	PAV (2021)	SVIM-asm (2020)	Dipcall (2018)
Total Benchmark Calls (>50): 4,116					
ONT_L1	**comp cnt**	5,631	5,660	5,785	7,284
	**TP-comp**	3,888	3,757	3,883	3,773
	**FP**	268	356	355	565
	**FN**	228	359	233	343
	**precision**	93.55%	91.34%	91.62%	86.98%
	**recall**	94.46%	91.28%	94.34%	91.67%
	**F1**	94.0%	91.31%	92.96%	89.26%
	**base cnt**	4,116	4,116	4,116	4,116
	**TP-comp_TP-gt**	3,856	3,709	3,836	3,521
	**TP-comp_FP-gt**	32	48	47	252
	**gt_concordance**	99.18%	98.72%	98.79%	93.32%
ONT_L2	**comp cnt**	5,493	5,793	5,778	6,967
	**TP-comp**	3,812	3,872	3,862	3,656
	**FP**	344	424	426	713
	**FN**	304	244	254	460
	**precision**	91.72%	90.13%	90.07%	83.68%
	**recall**	92.61%	94.07%	93.83%	88.82%
	**F1**	92.17%	92.06%	91.91%	86.18%
	**base cnt**	4,116	4,116	4,116	4,116
	**TP-comp_TP-gt**	3,778	3,811	3,801	3,267
	**TP-comp_FP-gt**	34	61	61	389
	**gt_concordance**	99.11%	98.42%	98.42%	89.36%
ONT_L3	**comp cnt**	5,655	5,691	5,780	7,216
	**TP-comp**	3,867	3,819	3,865	3,734
	**FP**	299	373	389	584
	**FN**	249	297	251	382
	**precision**	92.82%	91.1%	90.86%	86.48%
	**recall**	93.95%	92.78%	93.9%	90.72%
	**F1**	93.38%	91.94%	92.35%	88.55%
	**base cnt**	4,116	4,116	4,116	4,116
	**TP-comp_TP-gt**	3,829	3,771	3,818	3,459
	**TP-comp_FP-gt**	38	48	47	275
	**gt_concordance**	99.02%	98.74%	98.78%	92.64%
ONT_L4	**comp cnt**	5,680	5,846	5,806	6,205
	**TP-comp**	3,815	3,788	3,801	3,137
	**FP**	316	435	402	643
	**FN**	301	328	315	979
	**precision**	92.35%	89.7%	90.44%	82.99%
	**recall**	92.69%	92.03%	92.35%	76.21%
	**F1**	92.52%	90.85%	91.38%	79.46%
	**base cnt**	4,116	4,116	4,116	4,116
	**TP-comp_TP-gt**	3,772	3,678	3,690	2,698
	**TP-comp_FP-gt**	43	110	111	439
	**gt_concordance**	98.87%	97.1%	97.08%	86.01%
ONT_L5	**comp cnt**	5,596	5,563	5,768	7,182
	**TP-comp**	3,852	3,665	3,857	3,647
	**FP**	298	368	379	661
	**FN**	264	451	259	469
	**precision**	92.82%	90.88%	91.05%	84.66%
	**recall**	93.59%	89.04%	93.71%	88.61%
	**F1**	93.2%	89.95%	92.36%	86.59%
	**base cnt**	4,116	4,116	4,116	4,116
	**TP-comp_TP-gt**	3,817	3,618	3,808	3,289
	**TP-comp_FP-gt**	35	47	49	358
	**gt_concordance**	99.09%	98.72%	98.73%	90.18%
ONT_L6	**comp cnt**	5,562	5,651	5,793	7,131
	**TP-comp**	3,858	3,741	3,857	3,669
	**FP**	277	372	380	628
	**FN**	258	375	259	447
	**precision**	93.3%	90.96%	91.03%	85.39%
	**recall**	93.73%	90.89%	93.71%	89.14%
	**F1**	93.52%	90.92%	92.35%	87.22%
	**base cnt**	4,116	4,116	4,116	4,116
	**TP-comp_TP-gt**	3,822	3,694	3,811	3,341
	**TP-comp_FP-gt**	36	47	46	328
	**gt_concordance**	99.07%	98.74%	98.81%	91.06%

Supplementary Table 3: Genome-wide SV (≥50bp) evaluation against GIAB benchmark on **six ONT** datasets (p=0.5, P=0.5, r=500, and O=0.01 for Truvari). The left and right panels are for insertions (INS) and deletions (DEL), respectively. The evaluation metrics are true positive (TP-comp), false positive (FP), false negative (FN), precision, recall, F1, true positive for genotype accuracy (TP-comp_TP-gt), false positive for genotype accuracy (TP-comp_FP-gt), and genotype accuracy (gt_concordance). For TP-comp, precision, recall, F1, TP-comp_TP-gt, and gt_concordance, the highest values across all tools are highlighted in green color. The evaluated assembly-based tools are **VolcanoSV**, PAV, SVIM-asm, and Dipcall. Source data are provided as a Source Data file.

**Total**	Hifi_L1	Hifi_L2	Hifi_L3	Hifi_L4	Hifi_L5	CLR_L1	CLR_L2	CLR_L3	ONT_L1	ONT_L2	ONT_L3	ONT_L4	ONT_L5	ONT_L6
Unique TPs with correct GT #	300	227	213	223	241	402	1014	1125	183	249	199	477	253	219
Novel variants #	266	190	173	181	198	369	887	976	153	204	169	370	215	180
Existing variants #	34	37	40	42	43	33	127	149	30	45	30	107	38	39
Overlapped genes #	258	212	194	194	206	331	831	867	150	207	180	369	214	186
Overlapped transcripts #	939	836	737	723	740	1230	3162	3411	707	889	770	1513	889	791
Overlapped regulatory features #	120	57	54	56	72	157	314	304	54	81	72	144	68	74
Exact match with gnomAD #	29	29	35	31	39	26	110	125	24	34	24	89	35	33
Exact match with gnomAD (AF <=1%) #	12	7	8	8	9	7	49	56	8	13	5	39	12	12
Phased genes (SV) #	85	46	43	55	54	177	311	385	88	92	91	176	102	103
**DEL**	Hifi_L1	Hifi_L2	Hifi_L3	Hifi_L4	Hifi_L5	CLR_L1	CLR_L2	CLR_L3	ONT_L1	ONT_L2	ONT_L3	ONT_L4	ONT_L5	ONT_L6
Unique TPs with correct GT #	141	65	73	69	82	203	479	532	77	82	79	202	93	85
Novel variants #	116	38	39	39	49	179	401	431	51	57	57	135	68	60
Existing variants #	25	27	34	30	33	24	78	101	26	25	22	67	25	25
Overlapped genes #	117	71	72	63	66	163	392	409	62	73	73	144	82	73
Overlapped transcripts #	415	268	267	221	227	608	1498	1608	294	304	325	618	351	317
Overlapped regulatory features #	88	28	23	26	40	111	226	199	36	45	47	89	42	46
coding_sequence_variant %	89%	N/A	N/A	N/A	N/A	93%	67%	89%	N/A	20%	N/A	N/A	N/A	N/A
inframe_deletion %	11%	100%	100%	100%	100%	7%	33%	11%	100%	80%	100%	100%	100%	100%
frameshift_variant %	N/A	N/A	N/A	N/A	N/A	N/A	N/A	N/A	N/A	N/A	N/A	N/A	N/A	N/A
stop_retained_variant %	N/A	N/A	N/A	N/A	N/A	N/A	N/A	N/A	N/A	N/A	N/A	N/A	N/A	N/A
stop_gained %	N/A	N/A	N/A	N/A	N/A	N/A	N/A	N/A	N/A	N/A	N/A	N/A	N/A	N/A
Exact match with gnomAD #	25	24	32	28	35	22	83	95	23	25	20	67	25	23
Exact match with gnomAD (AF <=1%) #	8	5	6	7	7	6	34	37	7	9	4	31	8	8
**INS**	Hifi_L1	Hifi_L2	Hifi_L3	Hifi_L4	Hifi_L5	CLR_L1	CLR_L2	CLR_L3	ONT_L1	ONT_L2	ONT_L3	ONT_L4	ONT_L5	ONT_L6
Unique TPs with correct GT #	159	162	140	154	159	199	535	593	106	167	120	275	160	134
Novel variants #	150	152	134	142	149	190	486	545	102	147	112	235	147	120
Existing variants #	9	10	6	12	10	9	49	48	4	20	8	40	13	14
Overlapped genes #	141	141	122	131	140	168	439	458	88	134	107	225	132	113
Overlapped transcripts #	524	568	470	502	513	622	1664	1803	413	585	445	895	538	474
Overlapped regulatory features #	32	29	31	30	32	46	88	105	18	36	25	55	26	28
coding_sequence_variant %	N/A	N/A	N/A	N/A	N/A	N/A	N/A	N/A	N/A	N/A	N/A	N/A	N/A	N/A
inframe_deletion %	N/A	N/A	N/A	N/A	N/A	N/A	N/A	N/A	N/A	N/A	N/A	N/A	N/A	N/A
frameshift_variant %	N/A	N/A	N/A	N/A	N/A	N/A	100%	50%	N/A	N/A	N/A	N/A	N/A	N/A
stop_retained_variant %	N/A	N/A	N/A	N/A	N/A	N/A	N/A	50%	N/A	N/A	N/A	N/A	N/A	N/A
stop_gained %	N/A	N/A	N/A	N/A	N/A	N/A	N/A	N/A	N/A	N/A	N/A	100%	N/A	N/A
Exact match with gnomAD #	4	5	3	3	4	4	27	30	1	9	4	22	10	10
Exact match with gnomAD (AF <=1%) #	4	2	2	1	2	1	15	19	1	4	1	8	4	4

Supplementary Table 4: SV annotation using the Ensembl Variant Effect Predictor (VEP) and Genome Aggregation Database (gnomAD) for all datasets. “Unique TPs with correct GT” refer to additional true positive (TP) SVs with correct genotypes (GT) detected by VolcanoSV compared to the second-ranked tool in each dataset. Metrics from VEP based on these unique TPs with correct GT include “Novel variants”, “Phased genes”, “Existing variants”, “Overlapped genes”, “Overlapped transcripts”, and “Overlapped regulatory features”. “Exact match with gnomAD” indicates unique TP SVs with an exact match in terms of sequence and breakpoints with those in the gnomAD. AF stands for allele frequencies. “Phased genes” are additional genes phased by these unique TPs with correct GT, where genes are considered phased if all heterozygous variants within them are phased. The top, middle, and bottom panels are for total SVs, deletions (DELs), and insertions (INSs), respectively. Source data are provided as a Source Data file.

Total	Hifi_L2 Unique	CLR_L1 Unique	ONT_L1 Unique
Unique TPs #	43	174	56
Novel variants #	34	166	48
Existing variants #	9	8	8
Overlapped genes #	45	143	45
Overlapped transcripts #	130	544	263
Overlapped regulatory features #	17	104	19
Exact match with gnomAD #	9	8	6
Exact match with gnomAD (AF <=1%) #	3	4	3
**DEL**	Hifi_L2 Unique	CLR_L1 Unique	ONT_L1 Unique
Unique TPs with correct GT #	18	135	29
Novel variants #	12	129	22
Existing variants #	6	6	7
Overlapped genes #	26	113	21
Overlapped transcripts #	76	402	124
Overlapped regulatory features #	13	93	13
coding_sequence_variant %	N/A	100%	N/A
inframe_deletion %	N/A	N/A	N/A
frameshift_variant %	N/A	N/A	N/A
stop_retained_variant %	N/A	N/A	N/A
stop_gained %	N/A	N/A	N/A
Exact match with gnomAD #	5	6	5
Exact match with gnomAD (AF <=1%) #	1	3	2
**INS**	Hifi_L2 Unique	CLR_L1 Unique	ONT_L1 Unique
Unique TPs #	25	39	27
Novel variants #	22	37	26
Existing variants #	3	2	1
Overlapped genes #	19	30	24
Overlapped transcripts #	54	142	139
Overlapped regulatory features #	4	11	6
coding_sequence_variant %	N/A	N/A	N/A
inframe_deletion %	N/A	N/A	N/A
frameshift_variant %	N/A	N/A	N/A
stop_retained_variant %	N/A	N/A	N/A
stop_gained %	N/A	N/A	N/A
Exact match with gnomAD #	4	2	1
Exact match with gnomAD (AF <=1%) #	2	1	1

Supplementary Table 5: SV annotation using the Ensembl Variant Effect Predictor (VEP) and Genome Aggregation Database (gnomAD) for three representative datasets. “Unique TPs” refer to unique true positive (TP) SVs detected by VolcanoSV compared to PAV, SVIM-asm, and Dipcall in Hifi_L2, CLR_L1, ONT_L1. Metrics from VEP based on these unique TPs include “Novel variants”, “Phased genes”, “Existing variants”, “Overlapped genes”, “Overlapped transcripts”, and “Overlapped regulatory features”. “Exact match with gnomAD” indicates unique TP SVs with an exact match in terms of sequence and breakpoints with those in the gnomAD. AF stands for allele frequencies. “Phased genes” are additional genes phased by these unique TP SVs, where genes are considered phased if all heterozygous variants within them are phased. The top, middle, and bottom panels are for total SVs, deletions (DELs), and insertions (INSs), respectively. Source data are provided as a Source Data file.

	INS	VolcanoSV (2023)	PAV (2021)	SVIM-asm (2020)	Dipcall (2018)
Total Benchmark Calls (>50): 5,281					
	comp cnt	2,081	2,456	2,889	2,210
	TP-comp	2,539	2,407	2,847	2,169
	FP	340	623	720	790
	FN	2,742	2,874	2,434	3,112
	precision	88.19%	79.44%	79.81%	73.3%
5x	recall	48.08%	45.58%	53.91%	41.07%
	F1	62.23%	57.92%	64.35%	52.65%
	base cnt	5,281	5,281	5,281	5,281
	TP-comp_TP-gt	2,302	1,877	2,202	1,502
	TP-comp_FP-gt	237	530	645	667
	gt_concordance	90.67%	77.98%	77.34%	69.25%
	comp cnt	3,834	3,600	4,189	3,460
	TP-comp	4,608	4,159	4,497	3,787
	FP	595	683	1,026	1,509
	FN	673	1,122	784	1,494
	precision	88.56%	85.89%	81.42%	71.51%
10x	recall	87.26%	78.75%	85.15%	71.71%
	F1	87.91%	82.17%	83.25%	71.61%
	base cnt	5,281	5,281	5,281	5,281
	TP-comp_TP-gt	4,399	3,646	3,682	2,694
	TP-comp_FP-gt	209	513	815	1,093
	gt_concordance	95.46%	87.67%	81.88%	71.14%
	comp cnt	4,122	4,209	4,231	4,304
	TP-comp	4,915	4,930	4,925	4,823
	FP	641	743	796	1,913
	FN	366	351	356	458
	precision	88.46%	86.9%	86.09%	71.6%
20x	recall	93.07%	93.35%	93.26%	91.33%
	F1	90.71%	90.01%	89.53%	80.27%
	base cnt	5,281	5,281	5,281	5,281
	TP-comp_TP-gt	4,807	4,808	4,818	3,625
	TP-comp_FP-gt	108	122	107	1,198
	gt_concordance	97.8%	97.53%	97.83%	75.16%
	comp cnt	4,160	4,021	4,244	4,308
	TP-comp	4,957	4,737	4,957	4,851
	FP	657	744	814	1,944
	FN	324	544	324	430
	precision	88.3%	86.43%	85.89%	71.39%
30x	recall	93.86%	89.7%	93.86%	91.86%
	F1	91.0%	88.03%	89.7%	80.34%
	base cnt	5,281	5,281	5,281	5,281
	TP-comp_TP-gt	4,869	4,629	4,866	3,663
	TP-comp_FP-gt	88	108	91	1,188
	gt_concordance	98.22%	97.72%	98.16%	75.51%
	comp cnt	4,169	3,898	4,244	4,316
	TP-comp	4,961	4,583	4,966	4,864
	FP	657	725	806	1,949
	FN	320	698	315	417
	precision	88.31%	86.34%	86.04%	71.39%
40x	recall	93.94%	86.78%	94.04%	92.1%
	F1	91.04%	86.56%	89.86%	80.44%
	base cnt	5,281	5,281	5,281	5,281
	TP-comp_TP-gt	4,879	4,405	4,873	3,675
	TP-comp_FP-gt	82	178	93	1,189
	gt_concordance	98.35%	96.12%	98.13%	75.56%
	comp cnt	4,172	4,188	4,244	4,318
	TP-comp	4,965	4,944	4,959	4,852
	FP	647	758	801	1,915
	FN	316	337	322	429
	precision	88.47%	86.71%	86.09%	71.7%
50x	recall	94.02%	93.62%	93.9%	91.88%
	F1	91.16%	90.03%	89.83%	80.54%
	base cnt	5,281	5,281	5,281	5,281
	TP-comp_TP-gt	4,895	4,795	4,864	3,675
	TP-comp_FP-gt	70	149	95	1,177
	gt_concordance	98.59%	96.99%	98.08%	75.74%

	DEL	VolcanoSV (2023)	PAV (2021)	SVIM-asm (2020)	Dipcall (2018)
Total Benchmark Calls (>50): 4,116					
	comp cnt	2,879	3,030	3,567	2,959
	TP-comp	1,927	1,927	2,290	1,763
	FP	154	529	599	447
	FN	2,189	2,189	1,826	2,353
	precision	92.6%	78.46%	79.27%	79.77%
5x	recall	46.82%	46.82%	55.64%	42.83%
	F1	62.19%	58.64%	65.38%	55.74%
	base cnt	4,116	4,116	4,116	4,116
	TP-comp_TP-gt	1,783	1,383	1,626	1,276
	TP-comp_FP-gt	144	544	664	487
	gt_concordance	92.53%	71.77%	71.0%	72.38%
	comp cnt	5,203	4,842	5,523	5,296
	TP-comp	3,600	3,214	3,564	2,962
	FP	234	386	625	498
	FN	516	902	552	1,154
	precision	93.9%	89.28%	85.08%	85.61%
10x	recall	87.46%	78.09%	86.59%	71.96%
	F1	90.57%	83.31%	85.83%	78.19%
	base cnt	4,116	4,116	4,116	4,116
	TP-comp_TP-gt	3,523	2,731	2,766	2,575
	TP-comp_FP-gt	77	483	798	387
	gt_concordance	97.86%	84.97%	77.61%	86.93%
	comp cnt	5,556	5,673	5,721	6,736
	TP-comp	3,863	3,876	3,881	3,767
	FP	259	333	350	537
	FN	253	240	235	349
	precision	93.72%	92.09%	91.73%	87.52%
20x	recall	93.85%	94.17%	94.29%	91.52%
	F1	93.78%	93.12%	92.99%	89.48%
	base cnt	4,116	4,116	4,116	4,116
	TP-comp_TP-gt	3,816	3,796	3,793	3,608
	TP-comp_FP-gt	47	80	88	159
	gt_concordance	98.78%	97.94%	97.73%	95.78%
	comp cnt	5,614	5,481	5,771	6,795
	TP-comp	3,898	3,705	3,899	3,784
	FP	262	316	345	524
	FN	218	411	217	332
	precision	93.7%	92.14%	91.87%	87.84%
30x	recall	94.7%	90.01%	94.73%	91.93%
	F1	94.2%	91.07%	93.28%	89.84%
	base cnt	4,116	4,116	4,116	4,116
	TP-comp_TP-gt	3,859	3,646	3,835	3,649
	TP-comp_FP-gt	39	59	64	135
	gt_concordance	99.0%	98.41%	98.36%	96.43%
	comp cnt	5,618	5,308	5,772	6,813
	TP-comp	3,901	3,582	3,897	3,783
	FP	268	316	347	533
	FN	215	534	219	333
	precision	93.57%	91.89%	91.82%	87.65%
40x	recall	94.78%	87.03%	94.68%	91.91%
	F1	94.17%	89.39%	93.23%	89.73%
	base cnt	4,116	4,116	4,116	4,116
	TP-comp_TP-gt	3,866	3,487	3,837	3,651
	TP-comp_FP-gt	35	95	60	132
	gt_concordance	99.1%	97.35%	98.46%	96.51%
	comp cnt	5,612	5,702	5,760	6,767
	TP-comp	3,905	3,863	3,894	3,782
	FP	267	325	350	536
	FN	211	253	222	334
	precision	93.6%	92.24%	91.75%	87.59%
50x	recall	94.87%	93.85%	94.61%	91.89%
	F1	94.23%	93.04%	93.16%	89.68%
	base cnt	4,116	4,116	4,116	4,116
	TP-comp_TP-gt	3,869	3,782	3,828	3,650
	TP-comp_FP-gt	36	81	66	132
	gt_concordance	99.08%	97.9%	98.31%	96.51%

Supplementary Table 6: Genome-wide SV (≥50bp) evaluation against GIAB benchmark on **sub-sampled Hifi_L1** datasets which are at 5x, 10x, 20x, 30x, 40x, and 50x (p=0.5, P=0.5, r=500, and O=0.01 for Truvari). The left and right panels are for insertions (INS) and deletions (DEL), respectively. The evaluation metrics are true positive (TP-comp), false positive (FP), false negative (FN), precision, recall, F1, true positive for genotype accuracy (TP-comp_TP-gt), false positive for genotype accuracy (TP-comp_FP-gt), and genotype accuracy (gt_concordance). For TP-comp, precision, recall, F1, TP-comp_TP-gt, and gt_concordance, the highest values across all tools are highlighted in green color. The evaluated assembly-based tools are **VolcanoSV**, PAV, SVIM-asm, and Dipcall. Source data are provided as a Source Data file.

	INS	VolcanoSV (2023)	PAV (2021)	SVIM-asm (2020)	Dipcall (2018)
Total Benchmark Calls (>50): 5,281					
5x	comp cnt	1,038	5,224	2,481	32
	TP-comp	1,362	2,876	2,637	41
	FP	316	21,177	5,469	51
	FN	3,919	2,405	2,644	5,240
	precision	81.17%	11.96%	32.53%	44.57%
	recall	25.79%	54.46%	49.93%	0.78%
	F1	39.14%	19.61%	39.4%	1.53%
	base cnt	5,281	5,281	5,281	5,281
	TP-comp_TP-gt	1,158	1,994	1,849	29
	TP-comp_FP-gt	204	882	788	12
	gt_concordance	85.02%	69.33%	70.12%	70.73%
10x	comp cnt	2,980	4,344	3,176	31
	TP-comp	3,573	4,083	3,984	45
	FP	962	6,167	3,124	85
	FN	1,708	1,198	1,297	5,236
	precision	78.79%	39.83%	56.05%	34.62%
	recall	67.66%	77.31%	75.44%	0.85%
	F1	72.8%	52.58%	64.32%	1.66%
	base cnt	5,281	5,281	5,281	5,281
	TP-comp_TP-gt	3,266	3,083	2,974	25
	TP-comp_FP-gt	307	1,000	1,010	20
	gt_concordance	91.41%	75.51%	74.65%	55.56%
20x	comp cnt	4,065	4,122	3,718	803
	TP-comp	4,800	4,700	4,657	763
	FP	1,182	2,710	1,819	531
	FN	481	581	624	4,518
	precision	80.24%	63.43%	71.91%	58.96%
	recall	90.89%	89.0%	88.18%	14.45%
	F1	85.23%	74.07%	79.22%	23.21%
	base cnt	5,281	5,281	5,281	5,281
	TP-comp_TP-gt	4,613	4,053	4,026	362
	TP-comp_FP-gt	187	647	631	401
	gt_concordance	96.1%	86.23%	86.45%	47.44%
30x	comp cnt	4,129	3,971	3,914	2,900
	TP-comp	4,929	4,764	4,856	2,898
	FP	937	1,559	1,308	1,922
	FN	352	517	425	2,383
	precision	84.03%	75.34%	78.78%	60.12%
	recall	93.33%	90.21%	91.95%	54.88%
	F1	88.44%	82.11%	84.86%	57.38%
	base cnt	5,281	5,281	5,281	5,281
	TP-comp_TP-gt	4,790	4,372	4,485	1,532
	TP-comp_FP-gt	139	392	371	1,366
	gt_concordance	97.18%	91.77%	92.36%	52.86%
40x	comp cnt	4,137	4,117	4,012	3,954
	TP-comp	4,949	4,939	4,914	4,029
	FP	790	1,263	1,125	2,583
	FN	332	342	367	1,252
	precision	86.23%	79.64%	81.37%	60.93%
	recall	93.71%	93.52%	93.05%	76.29%
	F1	89.82%	86.02%	86.82%	67.75%
	base cnt	5,281	5,281	5,281	5,281
	TP-comp_TP-gt	4,842	4,613	4,627	2,246
	TP-comp_FP-gt	107	326	287	1,783
	gt_concordance	97.84%	93.4%	94.16%	55.75%
50x	comp cnt	4,136	4,019	4,071	4,175
	TP-comp	4,955	4,824	4,951	4,431
	FP	779	1,119	994	2,636
	FN	326	457	330	850
	precision	86.41%	81.17%	83.28%	62.7%
	recall	93.83%	91.35%	93.75%	83.9%
	F1	89.97%	85.96%	88.21%	71.77%
	base cnt	5,281	5,281	5,281	5,281
	TP-comp_TP-gt	4,866	4,557	4,711	2,619
	TP-comp_FP-gt	89	267	240	1,812
	gt_concordance	98.2%	94.47%	95.15%	59.11%

	DEL	VolcanoSV (2023)	PAV (2021)	SVIM-asm (2020)	Dipcall (2018)
Total Benchmark Calls (>50): 4,116					
5x	comp cnt	1,678	24,053	8,106	92
	TP-comp	962	2,005	1,829	25
	FP	76	3,219	652	7
	FN	3,154	2,111	2,287	4,091
	precision	92.68%	38.38%	73.72%	78.12%
	recall	23.37%	48.71%	44.44%	0.61%
	F1	37.33%	42.93%	55.45%	1.21%
	base cnt	4,116	4,116	4,116	4,116
	TP-comp_TP-gt	839	1,242	1,174	14
	TP-comp_FP-gt	123	763	655	11
	gt_concordance	87.21%	61.95%	64.19%	56.0%
10x	comp cnt	4,535	10,250	7,108	130
	TP-comp	2,764	2,897	2,839	24
	FP	216	1,447	337	7
	FN	1,352	1,219	1,277	4,092
	precision	92.75%	66.69%	89.39%	77.42%
	recall	67.15%	70.38%	68.97%	0.58%
	F1	77.9%	68.49%	77.87%	1.16%
	base cnt	4,116	4,116	4,116	4,116
	TP-comp_TP-gt	2,594	2,196	2,140	15
	TP-comp_FP-gt	170	701	699	9
	gt_concordance	93.85%	75.8%	75.38%	62.5%
20x	comp cnt	5,982	7,410	6,476	1,294
	TP-comp	3,791	3,357	3,320	552
	FP	274	765	398	251
	FN	325	759	796	3,564
	precision	93.26%	81.44%	89.3%	68.74%
	recall	92.1%	81.56%	80.66%	13.41%
	F1	92.68%	81.5%	84.76%	22.44%
	base cnt	4,116	4,116	4,116	4,116
	TP-comp_TP-gt	3,710	3,138	3,078	436
	TP-comp_FP-gt	81	219	242	116
	gt_concordance	97.86%	93.48%	92.71%	78.99%
30x	comp cnt	5,866	6,323	6,164	4,820
	TP-comp	3,870	3,435	3,556	2,135
	FP	259	536	358	765
	FN	246	681	560	1,981
	precision	93.73%	86.5%	90.85%	73.62%
	recall	94.02%	83.45%	86.39%	51.87%
	F1	93.88%	84.95%	88.57%	60.86%
	base cnt	4,116	4,116	4,116	4,116
	TP-comp_TP-gt	3,818	3,335	3,452	1,791
	TP-comp_FP-gt	52	100	104	344
	gt_concordance	98.66%	97.09%	97.08%	83.89%
40x	comp cnt	5,739	6,202	6,039	6,612
	TP-comp	3,886	3,662	3,643	3,004
	FP	251	455	369	950
	FN	230	454	473	1,112
	precision	93.93%	88.95%	90.8%	75.97%
	recall	94.41%	88.97%	88.51%	72.98%
	F1	94.17%	88.96%	89.64%	74.45%
	base cnt	4,116	4,116	4,116	4,116
	TP-comp_TP-gt	3,845	3,596	3,583	2,613
	TP-comp_FP-gt	41	66	60	391
	gt_concordance	98.94%	98.2%	98.35%	86.98%
50x	comp cnt	5,734	5,943	5,945	7,067
	TP-comp	3,890	3,589	3,701	3,321
	FP	246	430	370	854
	FN	226	527	415	795
	precision	94.05%	89.3%	90.91%	79.54%
	recall	94.51%	87.2%	89.92%	80.69%
	F1	94.28%	88.24%	90.41%	80.11%
	base cnt	4,116	4,116	4,116	4,116
	TP-comp_TP-gt	3,850	3,542	3,650	2,992
	TP-comp_FP-gt	40	47	51	329
	gt_concordance	98.97%	98.69%	98.62%	90.09%

Supplementary Table 7: Genome-wide SV (≥50bp) evaluation against GIAB benchmark on **subsampled CLR_L1** datasets which are at 5x, 10x, 20x, 30x, 40x, and 50x (p=0.5, P=0.5, r=500, and O=0.01 for Truvari). The left and right panels are for insertions (INS) and deletions (DEL), respectively. The evaluation metrics are true positive (TP-comp), false positive (FP), false negative (FN), precision, recall, F1, true positive for genotype accuracy (TP-comp_TP-gt), false positive for genotype accuracy (TP-comp_FP-gt), and genotype accuracy (gt_concordance). For TP-comp, precision, recall, F1, TP-comp_TP-gt, and gt_concordance, the highest values across all tools are highlighted in green color. The evaluated assembly-based tools are **VolcanoSV**, PAV, SVIM-asm, and Dipcall. Source data are provided as a Source Data file.

INS		VolcanoSV (2023)	PAV (2021)	SVIM-asm (2020)	Dipcall (2018)
Total Benchmark Calls (>50): 5,281					
5x	**comp cnt**	1,335	3,521	2,875	675
	**TP-comp**	1,762	3,455	3,334	665
	**FP**	313	1,365	991	408
	**FN**	3,519	1,826	1,947	4,616
	**precision**	84.92%	71.68%	77.09%	61.98%
	**recall**	33.36%	65.42%	63.13%	12.59%
	**F1**	47.91%	68.41%	69.41%	20.93%
	**base cnt**	5,281	5,281	5,281	5,281
	**TP-comp_TP-gt**	1,499	2,406	2,343	321
	**TP-comp_FP-gt**	263	1,049	991	344
	**gt_concordance**	85.07%	69.64%	70.28%	48.27%
10x	**comp cnt**	3,369	3,617	3,518	2,243
	**TP-comp**	4,057	4,183	4,135	2,036
	**FP**	715	936	894	1,460
	**FN**	1,224	1,098	1,146	3,245
	**precision**	85.02%	81.72%	82.22%	58.24%
	**recall**	76.82%	79.21%	78.3%	38.55%
	**F1**	80.71%	80.44%	80.21%	46.39%
	**base cnt**	5,281	5,281	5,281	5,281
	**TP-comp_TP-gt**	3,746	3,372	3,393	998
	**TP-comp_FP-gt**	311	811	742	1,038
	**gt_concordance**	92.33%	80.61%	82.06%	49.02%
20x	**comp cnt**	4,097	4,001	4,144	4,142
	**TP-comp**	4,841	4,587	4,745	3,965
	**FP**	725	952	972	2,487
	**FN**	440	694	536	1,316
	**precision**	86.97%	82.81%	83.0%	61.45%
	**recall**	91.67%	86.86%	89.85%	75.08%
	**F1**	89.26%	84.79%	86.29%	67.59%
	**base cnt**	5,281	5,281	5,281	5,281
	**TP-comp_TP-gt**	4,693	4,328	4,543	2,206
	**TP-comp_FP-gt**	148	259	202	1,759
	**gt_concordance**	96.94%	94.35%	95.74%	55.64%
30x	**comp cnt**	4,143	4,110	4,241	4,524
	**TP-comp**	4,914	4,777	4,903	4,600
	**FP**	696	886	911	2,733
	**FN**	367	504	378	681
	**precision**	87.59%	84.35%	84.33%	62.73%
	**recall**	93.05%	90.46%	92.84%	87.1%
	**F1**	90.24%	87.3%	88.38%	72.93%
	**base cnt**	5,281	5,281	5,281	5,281
	**TP-comp_TP-gt**	4,813	4,599	4,790	2,671
	**TP-comp_FP-gt**	101	178	113	1,929
	**gt_concordance**	97.94%	96.27%	97.7%	58.07%
40x	**comp cnt**	4,149	4,121	4,242	4,525
	**TP-comp**	4,937	4,821	4,939	4,730
	**FP**	700	845	857	2,646
	**FN**	344	460	342	551
	**precision**	87.58%	85.09%	85.21%	64.13%
	**recall**	93.49%	91.29%	93.52%	89.57%
	**F1**	90.44%	88.08%	89.18%	74.74%
	**base cnt**	5,281	5,281	5,281	5,281
	**TP-comp_TP-gt**	4,857	4,688	4,837	2,886
	**TP-comp_FP-gt**	80	133	102	1,844
	**gt_concordance**	98.38%	97.24%	97.93%	61.01%

DEL		VolcanoSV (2023)	PAV (2021)	SVIM-asm (2020)	Dipcall (2018)
Total Benchmark Calls (>50): 4,116					
5x	**comp cnt**	2,075	4,820	4,325	1,073
	**TP-comp**	1,225	2,427	2,364	503
	**FP**	110	1,094	511	172
	**FN**	2,891	1,689	1,752	3,613
	**precision**	91.76%	68.93%	82.23%	74.52%
	**recall**	29.76%	58.97%	57.43%	12.22%
	**F1**	44.95%	63.56%	67.63%	21.0%
	**base cnt**	4,116	4,116	4,116	4,116
	**TP-comp_TP-gt**	1,081	1,549	1,517	321
	**TP-comp_FP-gt**	144	878	847	182
	**gt_concordance**	88.24%	63.82%	64.17%	63.82%
10x	**comp cnt**	4,772	5,119	5,029	3,496
	**TP-comp**	3,117	3,146	3,156	1,573
	**FP**	252	471	362	670
	**FN**	999	970	960	2,543
	**precision**	92.52%	86.98%	89.71%	70.13%
	**recall**	75.73%	76.43%	76.68%	38.22%
	**F1**	83.29%	81.37%	82.68%	49.47%
	**base cnt**	4,116	4,116	4,116	4,116
	**TP-comp_TP-gt**	2,976	2,478	2,478	1,081
	**TP-comp_FP-gt**	141	668	678	492
	**gt_concordance**	95.48%	78.77%	78.52%	68.72%
20x	**comp cnt**	5,566	5,539	5,717	6,452
	**TP-comp**	3,793	3,603	3,757	3,094
	**FP**	304	398	387	1,048
	**FN**	323	513	359	1,022
	**precision**	92.58%	90.05%	90.66%	74.7%
	**recall**	92.15%	87.54%	91.28%	75.17%
	**F1**	92.37%	88.78%	90.97%	74.93%
	**base cnt**	4,116	4,116	4,116	4,116
	**TP-comp_TP-gt**	3,730	3,411	3,570	2,600
	**TP-comp_FP-gt**	63	192	187	494
	**gt_concordance**	98.34%	94.67%	95.02%	84.03%
30x	**comp cnt**	5,610	5,663	5,814	7,333
	**TP-comp**	3,862	3,732	3,858	3,556
	**FP**	281	378	383	968
	**FN**	254	384	258	560
	**precision**	93.22%	90.8%	90.97%	78.6%
	**recall**	93.83%	90.67%	93.73%	86.39%
	**F1**	93.52%	90.74%	92.33%	82.31%
	**base cnt**	4,116	4,116	4,116	4,116
	**TP-comp_TP-gt**	3,826	3,636	3,784	3,176
	**TP-comp_FP-gt**	36	96	74	380
	**gt_concordance**	99.07%	97.43%	98.08%	89.31%
40x	**comp cnt**	5,637	5,666	5,796	7,376
	**TP-comp**	3,873	3,757	3,877	3,644
	**FP**	276	364	365	881
	**FN**	243	359	239	472
	**precision**	93.35%	91.17%	91.4%	80.53%
	**recall**	94.1%	91.28%	94.19%	88.53%
	**F1**	93.72%	91.22%	92.77%	84.34%
	**base cnt**	4,116	4,116	4,116	4,116
	**TP-comp_TP-gt**	3,840	3,703	3,830	3,349
	**TP-comp_FP-gt**	33	54	47	295
	**gt_concordance**	99.15%	98.56%	98.79%	91.9%

Supplementary Table 8: Genome-wide SV ($\geq$50bp) evaluation against GIAB benchmark on **subsampled ONT_L1** datasets which are at 5x, 10x, 20x, 30x, 40x, and 50x (p=0.5, P=0.5, r=500, and O=0.01 for Truvari). The left and right panels are for insertions (INS) and deletions (DEL), respectively. The evaluation metrics are true positive (TP-comp), false positive (FP), false negative (FN), precision, recall, F1, true positive for genotype accuracy (TP-comp_TP-gt), false positive for genotype accuracy (TP-comp_FP-gt), and genotype accuracy (gt_concordance). For TP-comp, precision, recall, F1, TP-comp_TP-gt, and gt_concordance, the highest values across all tools are highlighted in green color. The evaluated assembly-based tools are **VolcanoSV**, PAV, SVIM-asm, and Dipcall. Source data are provided as a Source Data file.

INS		VolcanoSV-vc (2023)	PAV (2021)	SVIM-asm (2020)	Dipcall (2018)
Total Benchmark Calls (>50): 5,281					
Hifi_L1	comp cnt	4,134	4,158	4,215	4,220
	TP-comp	4,960	4,917	4,916	4,824
	FP	540	778	766	1,801
	FN	321	364	365	457
	precision	90.18%	86.34%	86.52%	72.82%
	recall	93.92%	93.11%	93.09%	91.35%
	F1	92.01%	89.6%	89.68%	81.03%
	base cnt	5,281	5,281	5,281	5,281
	TP-comp_TP-gt	4,888	4,699	4,809	3,755
	TP-comp_FP-gt	72	218	107	1,069
	gt_concordance	98.55%	95.57%	97.82%	77.84%
Hifi_L2	comp cnt	4,166	4,209	4,230	4,237
	TP-comp	4,966	4,929	4,907	4,837
	FP	594	779	774	1,776
	FN	315	352	374	444
	precision	89.32%	86.35%	86.38%	73.14%
	recall	94.04%	93.33%	92.92%	91.59%
	F1	91.62%	89.71%	89.53%	81.34%
	base cnt	5,281	5,281	5,281	5,281
	TP-comp_TP-gt	4,893	4,737	4,793	3,798
	TP-comp_FP-gt	73	192	114	1,039
	gt_concordance	98.53%	96.1%	97.68%	78.52%
Hifi_L3	comp cnt	4,164	4,196	4,239	4,245
	TP-comp	4,977	4,912	4,919	4,846
	FP	582	781	777	1,795
	FN	304	369	362	435
	precision	89.53%	86.28%	86.36%	72.97%
	recall	94.24%	93.01%	93.15%	91.76%
	F1	91.83%	89.52%	89.62%	81.3%
	base cnt	5,281	5,281	5,281	5,281
	TP-comp_TP-gt	4,912	4,661	4,818	3,794
	TP-comp_FP-gt	65	251	101	1,052
	gt_concordance	98.69%	94.89%	97.95%	78.29%
Hifi_L4	comp cnt	4,153	4,175	4,218	4,228
	TP-comp	4,973	4,911	4,921	4,832
	FP	588	788	769	1,795
	FN	308	370	360	449
	precision	89.43%	86.17%	86.49%	72.91%
	recall	94.17%	92.99%	93.18%	91.5%
	F1	91.74%	89.45%	89.71%	81.16%
	base cnt	5,281	5,281	5,281	5,281
	TP-comp_TP-gt	4,896	4,681	4,804	3,769
	TP-comp_FP-gt	77	230	117	1,063
	gt_concordance	98.45%	95.32%	97.62%	78.0%
Hifi_L5	comp cnt	4,157	4,230	4,233	4,225
	TP-comp	4,969	4,956	4,916	4,821
	FP	584	778	762	1,782
	FN	312	325	365	460
	precision	89.48%	86.43%	86.58%	73.01%
	recall	94.09%	93.85%	93.09%	91.29%
	F1	91.73%	89.99%	89.72%	81.13%
	base cnt	5,281	5,281	5,281	5,281
	TP-comp_TP-gt	4,885	4,807	4,797	3,768
	TP-comp_FP-gt	84	149	119	1,053
	gt_concordance	98.31%	96.99%	97.58%	78.16%

DEL		VolcanoSV-vc (2023)	PAV (2021)	SVIM-asm (2020)	Dipcall (2018)
Total Benchmark Calls (>50): 4,116					
Hifi_L1	comp cnt	5,500	5,695	5,682	6,625
	TP-comp	3,887	3,837	3,865	3,812
	FP	247	321	350	408
	FN	229	279	251	304
	precision	94.03%	92.28%	91.7%	90.33%
	recall	94.44%	93.22%	93.9%	92.61%
	F1	94.23%	92.75%	92.79%	91.46%
	base cnt	4,116	4,116	4,116	4,116
	TP-comp_TP-gt	3,830	3,726	3,782	3,688
	TP-comp_FP-gt	57	111	83	124
	gt_concordance	98.53%	97.11%	97.85%	96.75%
Hifi_L2	comp cnt	5,560	5,708	5,681	6,613
	TP-comp	3,903	3,874	3,892	3,818
	FP	263	335	338	419
	FN	213	242	224	298
	precision	93.69%	92.04%	92.01%	90.11%
	recall	94.83%	94.12%	94.56%	92.76%
	F1	94.25%	93.07%	93.27%	91.42%
	base cnt	4,116	4,116	4,116	4,116
	TP-comp_TP-gt	3,841	3,768	3,838	3,698
	TP-comp_FP-gt	62	106	54	120
	gt_concordance	98.41%	97.26%	98.61%	96.86%
Hifi_L3	comp cnt	5,559	5,693	5,696	6,641
	TP-comp	3,899	3,848	3,887	3,818
	FP	265	348	352	427
	FN	217	268	229	298
	precision	93.64%	91.71%	91.7%	89.94%
	recall	94.73%	93.49%	94.44%	92.76%
	F1	94.18%	92.59%	93.05%	91.33%
	base cnt	4,116	4,116	4,116	4,116
	TP-comp_TP-gt	3,837	3,718	3,832	3,697
	TP-comp_FP-gt	62	130	55	121
	gt_concordance	98.41%	96.62%	98.59%	96.83%
Hifi_L4	comp cnt	5,561	5,699	5,690	6,627
	TP-comp	3,893	3,845	3,886	3,811
	FP	260	330	332	417
	FN	223	271	230	305
	precision	93.74%	92.1%	92.13%	90.14%
	recall	94.58%	93.42%	94.41%	92.59%
	F1	94.16%	92.75%	93.26%	91.35%
	base cnt	4,116	4,116	4,116	4,116
	TP-comp_TP-gt	3,834	3,726	3,835	3,684
	TP-comp_FP-gt	59	119	51	127
	gt_concordance	98.48%	96.91%	98.69%	96.67%
Hifi_L5	comp cnt	5,553	5,734	5,678	6,603
	TP-comp	3,901	3,891	3,891	3,804
	FP	256	339	342	421
	FN	215	225	225	312
	precision	93.84%	91.99%	91.92%	90.04%
	recall	94.78%	94.53%	94.53%	92.42%
	F1	94.31%	93.24%	93.21%	91.21%
	base cnt	4,116	4,116	4,116	4,116
	TP-comp_TP-gt	3,840	3,838	3,835	3,686
	TP-comp_FP-gt	61	53	56	118
	gt_concordance	98.44%	98.64%	98.56%	96.9%

Supplementary Table 9: Genome-wide SV (≥50bp) evaluation against GIAB benchmark on **five Hifi** datasets (p=0.5, P=0.5, r=500, and O=0.01 for Truvari). The left and right panels are for insertions (INS) and deletions (DEL), respectively. The evaluation metrics are true positive (TP-comp), false positive (FP), false negative (FN), precision, recall, F1, true positive for genotype accuracy (TP-comp_TP-gt), false positive for genotype accuracy (TP-comp_FP-gt), and genotype accuracy (gt_concordance). For TP-comp, precision, recall, F1, TP-comp_TP-gt, and gt_concordance, the highest values across all tools are highlighted in green color. The evaluated assembly-based tools are **VolcanoSV-vc**, PAV, SVIM-asm, and Dipcall. Source data are provided as a Source Data file.

INS		VolcanoSV-vc (2023)	PAV (2021)	SVIM-asm (2020)	Dipcall (2018)
Total Benchmark Calls (>50): 5,281					
CLR_L1	**comp cnt**	3,993	4,017	4,103	4,030
	**TP-comp**	4,972	4,846	4,928	4,654
	**FP**	658	913	896	2,317
	**FN**	309	435	353	627
	**precision**	88.31%	84.15%	84.62%	66.76%
	**recall**	94.15%	91.76%	93.32%	88.13%
	**F1**	91.14%	87.79%	88.75%	75.97%
	**base cnt**	5,281	5,281	5,281	5,281
	**TP-comp_TP-gt**	4,816	4,646	4,769	3,146
	**TP-comp_FP-gt**	156	200	159	1,508
	**gt_concordance**	96.86%	95.87%	96.77%	67.6%
CLR_L2	**comp cnt**	3,874	4,387	4,004	1,051
	**TP-comp**	4,800	4,815	4,750	1,097
	**FP**	859	1,868	1,172	479
	**FN**	481	466	531	4,184
	**precision**	84.82%	72.05%	80.21%	69.61%
	**recall**	90.89%	91.18%	89.95%	20.77%
	**F1**	87.75%	80.49%	84.8%	32.0%
	**base cnt**	5,281	5,281	5,281	5,281
	**TP-comp_TP-gt**	4,501	4,418	4,370	746
	**TP-comp_FP-gt**	299	397	380	351
	**gt_concordance**	93.77%	91.75%	92.0%	68.0%
CLR_L3	**comp cnt**	3,734	3,931	3,829	2,296
	**TP-comp**	4,803	4,843	4,774	2,497
	**FP**	743	1,294	1,083	1,637
	**FN**	478	438	507	2,784
	**precision**	86.6%	78.91%	81.51%	60.4%
	**recall**	90.95%	91.71%	90.4%	47.28%
	**F1**	88.72%	84.83%	85.72%	53.04%
	**base cnt**	5,281	5,281	5,281	5,281
	**TP-comp_TP-gt**	4,355	4,322	4,290	1,355
	**TP-comp_FP-gt**	448	521	484	1,142
	**gt_concordance**	90.67%	89.24%	89.86%	54.27%

DEL		VolcanoSV-vc (2023)	PAV (2021)	SVIM-asm (2020)	Dipcall (2018)
Total Benchmark Calls (>50): 4,116					
CLR_L1	**comp cnt**	5,630	5,759	5,824	6,971
	**TP-comp**	3,753	3,664	3,775	3,579
	**FP**	240	353	328	451
	**FN**	363	452	341	537
	**precision**	93.99%	91.21%	92.01%	88.81%
	**recall**	91.18%	89.02%	91.72%	86.95%
	**F1**	92.56%	90.1%	91.86%	87.87%
	**base cnt**	4,116	4,116	4,116	4,116
	**TP-comp_TP-gt**	3,670	3,605	3,717	3,377
	**TP-comp_FP-gt**	83	59	58	202
	**gt_concordance**	97.79%	98.39%	98.46%	94.36%
CLR_L2	**comp cnt**	5,659	6,683	5,922	1,576
	**TP-comp**	3,567	3,576	3,544	866
	**FP**	307	811	460	185
	**FN**	549	540	572	3,250
	**precision**	92.08%	81.51%	88.51%	82.4%
	**recall**	86.66%	86.88%	86.1%	21.04%
	**F1**	89.29%	84.11%	87.29%	33.52%
	**base cnt**	4,116	4,116	4,116	4,116
	**TP-comp_TP-gt**	3,347	3,433	3,375	740
	**TP-comp_FP-gt**	220	143	169	126
	**gt_concordance**	93.83%	96.0%	95.23%	85.45%
CLR_L3	**comp cnt**	5,546	6,137	5,857	4,134
	**TP-comp**	3,452	3,472	3,469	1,907
	**FP**	282	459	360	389
	**FN**	664	644	647	2,209
	**precision**	92.45%	88.32%	90.6%	83.06%
	**recall**	83.87%	84.35%	84.28%	46.33%
	**F1**	87.95%	86.29%	87.33%	59.48%
	**base cnt**	4,116	4,116	4,116	4,116
	**TP-comp_TP-gt**	3,220	3,321	3,336	1,607
	**TP-comp_FP-gt**	232	151	133	300
	**gt_concordance**	93.28%	95.65%	96.17%	84.27%

Supplementary Table 10: Genome-wide SV (≥50bp) evaluation against GIAB benchmark on **three CLR** datasets (p=0.5, P=0.5, r=500, and O=0.01 for Truvari). The left and right panels are for insertions (INS) and deletions (DEL), respectively. The evaluation metrics are true positive (TP-comp), false positive (FP), false negative (FN), precision, recall, F1, true positive for genotype accuracy (TP-comp_TP-gt), false positive for genotype accuracy (TP-comp_FP-gt), and genotype accuracy (gt_concordance). For TP-comp, precision, recall, F1, TP-comp_TP-gt, and gt_concordance, the highest values across all tools are highlighted in green color. The evaluated assembly-based tools are **VolcanoSV-vc**, PAV, SVIM-asm, and Dipcall. Source data are provided as a Source Data file.

INS		VolcanoSV-vc (2023)	PAV (2021)	SVIM-asm (2020)	Dipcall (2018)	DEL		VolcanoSV-vc (2023)	PAV (2021)	SVIM-asm (2020)	Dipcall (2018)
Total Benchmark Calls (>50): 5,281						Total Benchmark Calls (>50): 4,116					
	**comp cnt**	4,163	4,113	4,238	4,338		**comp cnt**	5,559	5,660	5,785	7,284
	**TP-comp**	4,966	4,828	4,950	4,770		**TP-comp**	3,873	3,757	3,883	3,773
	**FP**	593	832	835	2,514		**FP**	290	356	355	565
	**FN**	315	453	331	511		**FN**	243	359	233	343
	**precision**	89.33%	85.3%	85.57%	65.49%		**precision**	93.03%	91.34%	91.62%	86.98%
ONT_L1	**recall**	94.04%	91.42%	93.73%	90.32%	ONT_L1	**recall**	94.1%	91.28%	94.34%	91.67%
	**F1**	91.62%	88.26%	89.46%	75.93%		**F1**	93.56%	91.31%	92.96%	89.26%
	**base cnt**	5,281	5,281	5,281	5,281		**base cnt**	4,116	4,116	4,116	4,116
	**TP-comp_TP-gt**	4,904	4,699	4,855	3,039		**TP-comp_TP-gt**	3,793	3,709	3,836	3,521
	**TP-comp_FP-gt**	62	129	95	1,731		**TP-comp_FP-gt**	80	48	47	252
	**gt_concordance**	98.75%	97.33%	98.08%	63.71%		**gt_concordance**	97.93%	98.72%	98.79%	93.32%
	**comp cnt**	4,214	4,296	4,288	4,369		**comp cnt**	5,501	5,793	5,778	6,967
	**TP-comp**	4,905	4,909	4,887	4,478		**TP-comp**	3,862	3,872	3,862	3,656
	**FP**	596	884	891	2,489		**FP**	352	424	426	713
	**FN**	376	372	394	803		**FN**	254	244	254	460
	**precision**	89.17%	84.74%	84.58%	64.27%		**precision**	91.65%	90.13%	90.07%	83.68%
ONT_L2	**recall**	92.88%	92.96%	92.54%	84.79%	ONT_L2	**recall**	93.83%	94.07%	93.83%	88.82%
	**F1**	90.98%	88.66%	88.38%	73.12%		**F1**	92.73%	92.06%	91.91%	86.18%
	**base cnt**	5,281	5,281	5,281	5,281		**base cnt**	4,116	4,116	4,116	4,116
	**TP-comp_TP-gt**	4,835	4,773	4,781	2,671		**TP-comp_TP-gt**	3,768	3,811	3,801	3,267
	**TP-comp_FP-gt**	70	136	106	1,807		**TP-comp_FP-gt**	94	61	61	389
	**gt_concordance**	98.57%	97.23%	97.83%	59.65%		**gt_concordance**	97.57%	98.42%	98.42%	89.36%
	**comp cnt**	4,163	4,192	4,254	4,318		**comp cnt**	5,574	5,691	5,780	7,216
	**TP-comp**	4,953	4,896	4,929	4,724		**TP-comp**	3,858	3,819	3,865	3,734
	**FP**	621	795	851	2,492		**FP**	305	373	389	584
	**FN**	328	385	352	557		**FN**	258	297	251	382
	**precision**	88.86%	86.03%	85.28%	65.47%		**precision**	92.67%	91.1%	90.86%	86.48%
ONT_L3	**recall**	93.79%	92.71%	93.33%	89.45%	ONT_L3	**recall**	93.73%	92.78%	93.9%	90.72%
	**F1**	91.26%	89.25%	89.12%	75.6%		**F1**	93.2%	91.94%	92.35%	88.55%
	**base cnt**	5,281	5,281	5,281	5,281		**base cnt**	4,116	4,116	4,116	4,116
	**TP-comp_TP-gt**	4,886	4,760	4,840	3,011		**TP-comp_TP-gt**	3,781	3,771	3,818	3,459
	**TP-comp_FP-gt**	67	136	89	1,713		**TP-comp_FP-gt**	77	48	47	275
	**gt_concordance**	98.65%	97.22%	98.19%	63.74%		**gt_concordance**	98.0%	98.74%	98.78%	92.64%
	**comp cnt**	4,124	4,223	4,203	3,780		**comp cnt**	5,512	5,846	5,806	6,205
	**TP-comp**	4,825	4,840	4,811	3,881		**TP-comp**	3,777	3,788	3,801	3,137
	**FP**	687	1,006	995	2,324		**FP**	347	435	402	643
	**FN**	456	441	470	1,400		**FN**	339	328	315	979
	**precision**	87.54%	82.79%	82.86%	62.55%		**precision**	91.59%	89.7%	90.44%	82.99%
ONT_L4	**recall**	91.37%	91.65%	91.1%	73.49%	ONT_L4	**recall**	91.76%	92.03%	92.35%	76.21%
	**F1**	89.41%	87.0%	86.79%	67.58%		**F1**	91.67%	90.85%	91.38%	79.46%
	**base cnt**	5,281	5,281	5,281	5,281		**base cnt**	4,116	4,116	4,116	4,116
	**TP-comp_TP-gt**	4,708	4,637	4,646	2,152		**TP-comp_TP-gt**	3,604	3,678	3,690	2,698
	**TP-comp_FP-gt**	117	203	165	1,729		**TP-comp_FP-gt**	173	110	111	439
	**gt_concordance**	97.58%	95.81%	96.57%	55.45%		**gt_concordance**	95.42%	97.1%	97.08%	86.01%
	**comp cnt**	4,165	4,033	4,236	4,308		**comp cnt**	5,574	5,563	5,768	7,182
	**TP-comp**	4,927	4,713	4,906	4,584		**TP-comp**	3,854	3,665	3,857	3,647
	**FP**	647	850	862	2,598		**FP**	311	368	379	661
	**FN**	354	568	375	697		**FN**	262	451	259	469
	**precision**	88.39%	84.72%	85.06%	63.83%		**precision**	92.53%	90.88%	91.05%	84.66%
ONT_L5	**recall**	93.3%	89.24%	92.9%	86.8%	ONT_L5	**recall**	93.63%	89.04%	93.71%	88.61%
	**F1**	90.78%	86.92%	88.8%	73.56%		**F1**	93.08%	89.95%	92.36%	86.59%
	**base cnt**	5,281	5,281	5,281	5,281		**base cnt**	4,116	4,116	4,116	4,116
	**TP-comp_TP-gt**	4,851	4,580	4,804	2,716		**TP-comp_TP-gt**	3,754	3,618	3,808	3,289
	**TP-comp_FP-gt**	76	133	102	1,868		**TP-comp_FP-gt**	100	47	49	358
	**gt_concordance**	98.46%	97.18%	97.92%	59.25%		**gt_concordance**	97.41%	98.72%	98.73%	90.18%
	**comp cnt**	4,172	4,113	4,237	4,297		**comp cnt**	5,557	5,651	5,793	7,131
	**TP-comp**	4,922	4,791	4,894	4,594		**TP-comp**	3,864	3,741	3,857	3,669
	**FP**	635	860	899	2,537		**FP**	308	372	380	628
	**FN**	359	490	387	687		**FN**	252	375	259	447
	**precision**	88.57%	84.78%	84.48%	64.42%		**precision**	92.62%	90.96%	91.03%	85.39%
ONT_L6	**recall**	93.2%	90.72%	92.67%	86.99%	ONT_L6	**recall**	93.88%	90.89%	93.71%	89.14%
	**F1**	90.83%	87.65%	88.39%	74.03%		**F1**	93.24%	90.92%	92.35%	87.22%
	**base cnt**	5,281	5,281	5,281	5,281		**base cnt**	4,116	4,116	4,116	4,116
	**TP-comp_TP-gt**	4,855	4,652	4,801	2,783		**TP-comp_TP-gt**	3,773	3,694	3,811	3,341
	**TP-comp_FP-gt**	67	139	93	1,811		**TP-comp_FP-gt**	91	47	46	328
	**gt_concordance**	98.64%	97.1%	98.1%	60.58%		**gt_concordance**	97.64%	98.74%	98.81%	91.06%

Supplementary Table 11: Genome-wide SV (≥50bp) evaluation against GIAB benchmark on **six ONT** datasets (p=0.5, P=0.5, r=500, and O=0.01 for Truvari). The left and right panels are for insertions (INS) and deletions (DEL), respectively. The evaluation metrics are true positive (TP-comp), false positive (FP), false negative (FN), precision, recall, F1, true positive for genotype accuracy (TP-comp_TP-gt), false positive for genotype accuracy (TP-comp_FP-gt), and genotype accuracy (gt_concordance). For TP-comp, precision, recall, F1, TP-comp_TP-gt, and gt_concordance, the highest values across all tools are highlighted in green color. The evaluated assembly-based tools are **VolcanoSV-vc**, PAV, SVIM-asm, and Dipcall. Source data are provided as a Source Data file.

	INDEL	VolcanoSV (2023)	PAV (2021)	Dipcall (2018)		SNP	VolcanoSV (2023)	PAV (2021)	Dipcall (2018)
Total Benchmark Calls (2-49 bp): 260,272					Total Benchmark Calls: 3,339,730				
Hifi_L1	comp cnt	262,797	268,801	260,359	Hifi_L1	comp cnt	3,303,306	3,304,133	3,321,998
	TP-comp	257,180	251,235	254,861		TP-comp	3,284,137	3,278,505	3,304,697
	FP	5,617	17,566	5,498		FP	19,169	25,628	17,301
	FN	3,092	9,037	5,411		FN	55,593	61,225	35,033
	precision	97.86%	93.47%	97.89%		precision	99.42%	99.22%	99.48%
	recall	98.81%	96.53%	97.92%		recall	98.34%	98.17%	98.95%
	F1	98.34%	94.97%	97.9%		F1	98.87%	98.69%	99.21%
	base cnt	260,272	260,272	260,272		base cnt	3,339,730	3,339,730	3,339,730
	TP-comp_TP-gt	247,668	213,497	249,916		TP-comp_TP-gt	3,283,671	3,215,977	3,298,656
	TP-comp_FP-gt	9,512	37,738	4,945		TP-comp_FP-gt	19,635	88,156	23,342
	gt_concordance	96.3%	84.98%	98.06%		gt_concordance	99.99%	98.09%	99.82%
Hifi_L2	comp cnt	263,140	268,740	259,825	Hifi_L2	comp cnt	3,347,870	3,314,760	3,326,494
	TP-comp	254,891	250,046	252,624		TP-comp	3,323,562	3,288,262	3,309,167
	FP	8,249	18,694	7,201		FP	24,308	26,498	17,327
	FN	5,381	10,226	7,648		FN	16,168	51,468	30,563
	precision	96.87%	93.04%	97.23%		precision	99.27%	99.2%	99.48%
	recall	97.93%	96.07%	97.06%		recall	99.52%	98.46%	99.08%
	F1	97.4%	94.53%	97.14%		F1	99.39%	98.83%	99.28%
	base cnt	260,272	260,272	260,272		base cnt	3,339,730	3,339,730	3,339,730
	TP-comp_TP-gt	241,705	212,373	245,138		TP-comp_TP-gt	3,322,935	3,236,356	3,304,516
	TP-comp_FP-gt	13,186	37,673	7,486		TP-comp_FP-gt	24,935	78,404	21,978
	gt_concordance	94.83%	84.93%	97.04%		gt_concordance	99.98%	98.42%	99.86%
Hifi_L3	comp cnt	262,898	267,522	260,391	Hifi_L3	comp cnt	3,345,798	3,300,150	3,325,692
	TP-comp	256,222	249,561	253,870		TP-comp	3,323,392	3,275,760	3,308,733
	FP	6,676	17,961	6,521		FP	22,406	24,390	16,959
	FN	4,050	10,711	6,402		FN	16,338	63,970	30,997
	precision	97.46%	93.29%	97.5%		precision	99.33%	99.26%	99.49%
	recall	98.44%	95.88%	97.54%		recall	99.51%	98.08%	99.07%
	F1	97.95%	94.57%	97.52%		F1	99.42%	98.67%	99.28%
	base cnt	260,272	260,272	260,272		base cnt	3,339,730	3,339,730	3,339,730
	TP-comp_TP-gt	245,417	211,204	247,534		TP-comp_TP-gt	3,322,799	3,202,942	3,305,157
	TP-comp_FP-gt	10,805	38,357	6,336		TP-comp_FP-gt	22,999	97,208	20,535
	gt_concordance	95.78%	84.63%	97.5%		gt_concordance	99.98%	97.78%	99.89%
Hifi_L4	comp cnt	265,787	270,445	262,083	Hifi_L4	comp cnt	3,348,949	3,298,538	3,323,978
	TP-comp	254,555	248,961	252,546		TP-comp	3,323,261	3,272,322	3,305,362
	FP	11,232	21,484	9,537		FP	25,688	26,216	18,616
	FN	5,717	11,311	7,726		FN	16,469	67,408	34,368
	precision	95.77%	92.06%	96.36%		precision	99.23%	99.21%	99.44%
	recall	97.8%	95.65%	97.03%		recall	99.51%	97.98%	98.97%
	F1	96.78%	93.82%	96.7%		F1	99.37%	98.59%	99.2%
	base cnt	260,272	260,272	260,272		base cnt	3,339,730	3,339,730	3,339,730
	TP-comp_TP-gt	241,915	210,421	244,495		TP-comp_TP-gt	3,322,613	3,202,224	3,301,119
	TP-comp_FP-gt	12,640	38,540	8,051		TP-comp_FP-gt	26,336	96,314	22,859
	gt_concordance	95.03%	84.52%	96.81%		gt_concordance	99.98%	97.86%	99.87%
Hifi_L5	comp cnt	263,205	272,976	260,950	Hifi_L5	comp cnt	3,346,012	3,335,170	3,317,849
	TP-comp	256,848	254,314	254,742		TP-comp	3,322,896	3,308,419	3,301,020
	FP	6,357	18,662	6,208		FP	23,116	26,751	16,829
	FN	3,424	5,958	5,530		FN	16,834	31,311	38,710
	precision	97.58%	93.16%	97.62%		precision	99.31%	99.2%	99.49%
	recall	98.68%	97.71%	97.88%		recall	99.5%	99.06%	98.84%
	F1	98.13%	95.38%	97.75%		F1	99.4%	99.13%	99.17%
	base cnt	260,272	260,272	260,272		base cnt	3,339,730	3,339,730	3,339,730
	TP-comp_TP-gt	248,189	219,300	249,114		TP-comp_TP-gt	3,322,270	3,303,911	3,299,616
	TP-comp_FP-gt	8,659	35,014	5,628		TP-comp_FP-gt	23,742	31,259	18,233
	gt_concordance	96.63%	86.23%	97.79%		gt_concordance	99.98%	99.86%	99.96%

Supplementary Table 12: Genome-wide snp and small indel (<50bp) evaluation against GIAB benchmark on **five Hifi** datasets (p=0.5, P=0.5, r=500, and O=0.01 for Truvari). The left and right panels are for small indels and SNPs, respectively. The evaluation metrics are true positive (TP-comp), false positive (FP), false negative (FN), precision, recall, F1, true positive for genotype accuracy (TP-comp_TP-gt), false positive for genotype accuracy (TP-comp_FP-gt), and genotype accuracy (gt_concordance). For TP-comp, precision, recall, F1, TP-comp_TP-gt, and gt_concordance, the highest values across all tools are highlighted in green color. The evaluated assembly-based tools are **VolcanoSV**, PAV, and Dipcall. Source data are provided as a Source Data file.

	INDEL	VolcanoSV (2023)	PAV (2021)	Dipcall (2018)		SNP	VolcanoSV (2023)	PAV (2021)	Dipcall (2018)
Total Benchmark Calls (2-49 bp): 260,272					Total Benchmark Calls: 3,339,730				
CLR_L1	comp cnt	265,008	455,525	408,849	CLR_L1	comp cnt	3,322,833	3,453,689	3,341,159
	TP-comp	251,173	236,681	244,140		TP-comp	3,283,219	3,197,550	3,253,685
	FP	13,835	218,844	164,709		FP	39,614	256,139	87,474
	FN	9,099	23,591	16,132		FN	56,511	142,180	86,045
	precision	94.78%	51.96%	59.71%		precision	98.81%	92.58%	97.38%
	recall	96.5%	90.94%	93.8%		recall	98.31%	95.74%	97.42%
	F1	95.63%	66.13%	72.97%		F1	98.56%	94.14%	97.4%
	base cnt	260,272	260,272	260,272		base cnt	3,339,730	3,339,730	3,339,730
	TP-comp_TP-gt	235,962	195,952	209,198		TP-comp_TP-gt	3,282,487	3,188,940	3,250,704
	TP-comp_FP-gt	15,211	40,729	34,942		TP-comp_FP-gt	40,346	264,749	90,455
	gt_concordance	93.94%	82.79%	85.69%		gt_concordance	99.98%	99.73%	99.91%
CLR_L2	comp cnt	247,085	3,705,755	843,108	CLR_L2	comp cnt	3,378,464	5,027,222	1,259,655
	TP-comp	211,696	233,443	135,374		TP-comp	3,323,992	3,229,568	1,054,941
	FP	35,389	3,472,312	707,734		FP	54,472	1,797,654	204,714
	FN	48,576	26,829	124,898		FN	15,738	110,162	2,284,789
	precision	85.68%	6.3%	16.06%		precision	98.39%	64.24%	83.75%
	recall	81.34%	89.69%	52.01%		recall	99.53%	96.7%	31.59%
	F1	83.45%	11.77%	24.54%		F1	98.95%	77.2%	45.87%
	base cnt	260,272	260,272	260,272		base cnt	3,339,730	3,339,730	3,339,730
	TP-comp_TP-gt	179,113	176,258	59,660		TP-comp_TP-gt	3,323,045	3,155,098	1,051,078
	TP-comp_FP-gt	32,583	57,185	75,714		TP-comp_FP-gt	55,419	1,872,124	208,577
	gt_concordance	84.61%	75.5%	44.07%		gt_concordance	99.97%	97.69%	99.63%
CLR_L3	comp cnt	144,700	2,321,618	1,295,362	CLR_L3	comp cnt	3,292,281	6,473,808	3,074,216
	TP-comp	124,745	237,794	205,438		TP-comp	3,239,242	3,225,581	2,021,878
	FP	19,955	2,083,824	1,089,924		FP	53,039	3,248,227	1,052,338
	FN	135,527	22,478	54,834		FN	100,488	114,149	1,317,852
	precision	86.21%	10.24%	15.86%		precision	98.39%	49.83%	65.77%
	recall	47.93%	91.36%	78.93%		recall	96.99%	96.58%	60.54%
	F1	61.61%	18.42%	26.41%		F1	97.69%	65.74%	63.05%
	base cnt	260,272	260,272	260,272		base cnt	3,339,730	3,339,730	3,339,730
	TP-comp_TP-gt	105,071	181,879	119,870		TP-comp_TP-gt	3,237,042	3,140,128	2,004,229
	TP-comp_FP-gt	19,674	55,915	85,568		TP-comp_FP-gt	55,239	3,333,680	1,069,987
	gt_concordance	84.23%	76.49%	58.35%		gt_concordance	99.93%	97.35%	99.13%

Supplementary Table 13: Genome-wide snp and small indel (<50bp) evaluation against GIAB benchmark on **three CLR** datasets (p=0.5, P=0.5, r=500, and O=0.01 for Truvari). The left and right panels are for small indels and SNPs, respectively. The evaluation metrics are true positive (TP-comp), false positive (FP), false negative (FN), precision, recall, F1, true positive for genotype accuracy (TP-comp_TP-gt), false positive for genotype accuracy (TP-comp_FP-gt), and genotype accuracy (gt_concordance). For TP-comp, precision, recall, F1, TP-comp_TP-gt, and gt_concordance, the highest values across all tools are highlighted in green color. The evaluated assembly-based tools are **VolcanoSV**, PAV, and Dipcall. Source data are provided as a Source Data file.

	INDEL	VolcanoSV (2023)	PAV (2021)	Dipcall (2018)
Total Benchmark Calls (2-49 bp): 260,272				
ONT_L1	comp cnt	691,737	767,601	761,755
	TP-comp	230,321	233,135	238,862
	FP	461,416	534,466	522,893
	FN	29,951	27,137	21,410
	precision	33.3%	30.37%	31.36%
	recall	88.49%	89.57%	91.77%
	F1	48.39%	45.36%	46.74%
	base cnt	260,272	260,272	260,272
	TP-comp_TP-gt	199,690	192,357	208,654
	TP-comp_FP-gt	30,631	40,778	30,208
	gt_concordance	86.7%	82.51%	87.35%
ONT_L2	comp cnt	932,495	2,381,279	3,210,076
	TP-comp	164,342	201,037	203,151
	FP	768,153	2,180,242	3,006,925
	FN	95,930	59,235	57,121
	precision	17.62%	8.44%	6.33%
	recall	63.14%	77.24%	78.05%
	F1	27.56%	15.22%	11.71%
	base cnt	260,272	260,272	260,272
	TP-comp_TP-gt	134,097	160,226	162,442
	TP-comp_FP-gt	30,245	40,811	40,709
	gt_concordance	81.6%	79.7%	79.96%
ONT_L3	comp cnt	937,206	994,061	1,008,887
	TP-comp	231,951	237,710	239,101
	FP	705,255	756,351	769,786
	FN	28,321	22,562	21,171
	precision	24.75%	23.91%	23.7%
	recall	89.12%	91.33%	91.87%
	F1	38.74%	37.9%	37.68%
	base cnt	260,272	260,272	260,272
	TP-comp_TP-gt	197,010	195,350	206,769
	TP-comp_FP-gt	34,941	42,360	32,332
	gt_concordance	84.94%	82.18%	86.48%
ONT_L4	comp cnt	861,564	2,619,513	2,476,190
	TP-comp	207,764	236,118	226,017
	FP	653,800	2,383,395	2,250,173
	FN	52,508	24,154	34,255
	precision	24.11%	9.01%	9.13%
	recall	79.83%	90.72%	86.84%
	F1	37.04%	16.4%	16.52%
	base cnt	260,272	260,272	260,272
	TP-comp_TP-gt	171,956	181,922	165,246
	TP-comp_FP-gt	35,808	54,196	60,771
	gt_concordance	82.77%	77.05%	73.11%
ONT_L5	comp cnt	1,008,842	1,401,912	1,514,656
	TP-comp	212,830	225,204	234,323
	FP	796,012	1,176,708	1,280,333
	FN	47,442	35,068	25,949
	precision	21.1%	16.06%	15.47%
	recall	81.77%	86.53%	90.03%
	F1	33.54%	27.1%	26.4%
	base cnt	260,272	260,272	260,272
	TP-comp_TP-gt	172,936	179,865	191,487
	TP-comp_FP-gt	39,894	45,339	42,836
	gt_concordance	81.26%	79.87%	81.72%
ONT_L6	comp cnt	1,052,494	1,312,149	1,395,163
	TP-comp	218,509	230,745	235,043
	FP	833,985	1,081,404	1,160,120
	FN	41,763	29,527	25,229
	precision	20.76%	17.59%	16.85%
	recall	83.95%	88.66%	90.31%
	F1	33.29%	29.35%	28.4%
	base cnt	260,272	260,272	260,272
	TP-comp_TP-gt	180,695	186,545	195,673
	TP-comp_FP-gt	37,814	44,200	39,370
	gt_concordance	82.69%	80.84%	83.25%

	SNP	VolcanoSV (2023)	PAV (2021)	Dipcall (2018)
Total Benchmark Calls: 3,339,730				
ONT_L1	comp cnt	3,387,086	3,735,675	3,745,923
	TP-comp	3,290,391	3,174,134	3,239,574
	FP	96,695	561,541	506,349
	FN	49,339	165,596	100,156
	precision	97.15%	84.97%	86.48%
	recall	98.52%	95.04%	97.0%
	F1	97.83%	89.72%	91.44%
	base cnt	3,339,730	3,339,730	3,339,730
	TP-comp_TP-gt	3,288,189	3,156,489	3,234,311
	TP-comp_FP-gt	98,897	579,186	511,612
	gt_concordance	99.93%	99.44%	99.84%
ONT_L2	comp cnt	3,433,723	4,429,269	4,451,049
	TP-comp	3,188,582	3,221,091	3,137,903
	FP	245,141	1,208,178	1,313,146
	FN	151,148	118,639	201,827
	precision	92.86%	72.72%	70.5%
	recall	95.47%	96.45%	93.96%
	F1	94.15%	82.92%	80.55%
	base cnt	3,339,730	3,339,730	3,339,730
	TP-comp_TP-gt	3,180,614	3,192,180	3,130,361
	TP-comp_FP-gt	253,109	1,237,089	1,320,688
	gt_concordance	99.75%	99.1%	99.76%
ONT_L3	comp cnt	3,358,958	3,565,083	3,569,326
	TP-comp	3,282,099	3,220,932	3,228,592
	FP	76,859	344,151	340,734
	FN	57,631	118,798	111,138
	precision	97.71%	90.35%	90.45%
	recall	98.27%	96.44%	96.67%
	F1	97.99%	93.3%	93.46%
	base cnt	3,339,730	3,339,730	3,339,730
	TP-comp_TP-gt	3,280,205	3,201,324	3,223,810
	TP-comp_FP-gt	78,753	363,759	345,516
	gt_concordance	99.94%	99.39%	99.85%
ONT_L4	comp cnt	3,496,404	7,871,942	6,657,737
	TP-comp	3,267,732	3,204,619	2,814,740
	FP	228,672	4,667,323	3,842,997
	FN	71,998	135,111	524,990
	precision	93.46%	40.71%	42.28%
	recall	97.84%	95.95%	84.28%
	F1	95.6%	57.17%	56.31%
	base cnt	3,339,730	3,339,730	3,339,730
	TP-comp_TP-gt	3,261,697	3,128,259	2,787,346
	TP-comp_FP-gt	234,707	4,743,683	3,870,391
	gt_concordance	99.82%	97.62%	99.03%
ONT_L5	comp cnt	3,456,707	4,344,939	4,474,749
	TP-comp	3,263,421	3,075,198	3,169,516
	FP	193,286	1,269,741	1,305,233
	FN	76,309	264,532	170,214
	precision	94.41%	70.78%	70.83%
	recall	97.72%	92.08%	94.9%
	F1	96.03%	80.03%	81.12%
	base cnt	3,339,730	3,339,730	3,339,730
	TP-comp_TP-gt	3,258,414	3,044,449	3,161,301
	TP-comp_FP-gt	198,293	1,300,490	1,313,448
	gt_concordance	99.85%	99.0%	99.74%
ONT_L6	comp cnt	3,433,425	4,131,828	4,176,890
	TP-comp	3,254,548	3,141,989	3,179,681
	FP	178,877	989,839	997,209
	FN	85,182	197,741	160,049
	precision	94.79%	76.04%	76.13%
	recall	97.45%	94.08%	95.21%
	F1	96.1%	84.11%	84.6%
	base cnt	3,339,730	3,339,730	3,339,730
	TP-comp_TP-gt	3,249,902	3,113,126	3,172,131
	TP-comp_FP-gt	183,523	1,018,702	1,004,759
	gt_concordance	99.86%	99.08%	99.76%

Supplementary Table 14: Genome-wide snp and small indel (<50bp) evaluation against GIAB benchmark on **six ONT** datasets (p=0.5, P=0.5, r=500, and O=0.01 for Truvari). The left and right panels are for small indels and SNPs, respectively. The evaluation metrics are true positive (TP-comp), false positive (FP), false negative (FN), precision, recall, F1, true positive for genotype accuracy (TP-comp_TP-gt), false positive for genotype accuracy (TP-comp_FP-gt), and genotype accuracy (gt_concordance). For TP-comp, precision, recall, F1, TP-comp_TP-gt, and gt_concordance, the highest values across all tools are highlighted in green color. The evaluated assembly-based tools are **VolcanoSV**, PAV, and Dipcall. Source data are provided as a Source Data file.

**Hifi_L1**				
tool	ncpu	memory(GB)	CPU hours	Run time
VolcanoSV-asm	30	168	1474	2-01:07:59
hifiasm	20	214	440	21:44:01

**CLR_L1**				
tool	ncpu	memory(GB)	CPU hours	Run time
VolcanoSV-asm	20	259	2582	5-09:05:59
flye+hapdup	50	691	10673	8-21:27:36

**ONT_L1**				
tool	ncpu	memory(GB)	CPU hours	Run time
VolcanoSV-asm	30	85	4397	6-02:33:59
flye+hapdup	64	337	7886	5-03:13:07

Supplementary Table 15: Computing resource consumption of different assemblers on **Hifi_L1**, **CLR_L1**, and **ONT_L1**.

**Hifi_L1**				
tool	ncpu	memory(GB)	CPU hours	Run time
VolcanoSV-vc	50	21	21	00:25:11
PAV	20	101	97	04:50:59
SVIM-asm	20	37	17	00:51:00
Dipcall	12	48	11	00:55:00

**CLR_L1**				
tool	ncpu	memory(GB)	CPU hours	Run time
VolcanoSV-vc	50	215	123	02:27:35
PAV	12	67	1098	3-19:31:55
SVIM-asm	10	26	7	00:42:00
Dipcall	10	42	10	01:00:00

**ONT_L1**				
tool	ncpu	memory(GB)	CPU hours	Run time
VolcanoSV-vc	50	34	32	00:38:24
PAV	10	53	133	13:18:00
SVIM-asm	20	34	8	00:24:00
Dipcall	12	47	8	00:40:00

Supplementary Table 16: Computing resource consumption of different assembly-based SV callers on **Hifi_L1**, **CLR_L1**, and **ONT_L1**.

3 Supplementary Figures

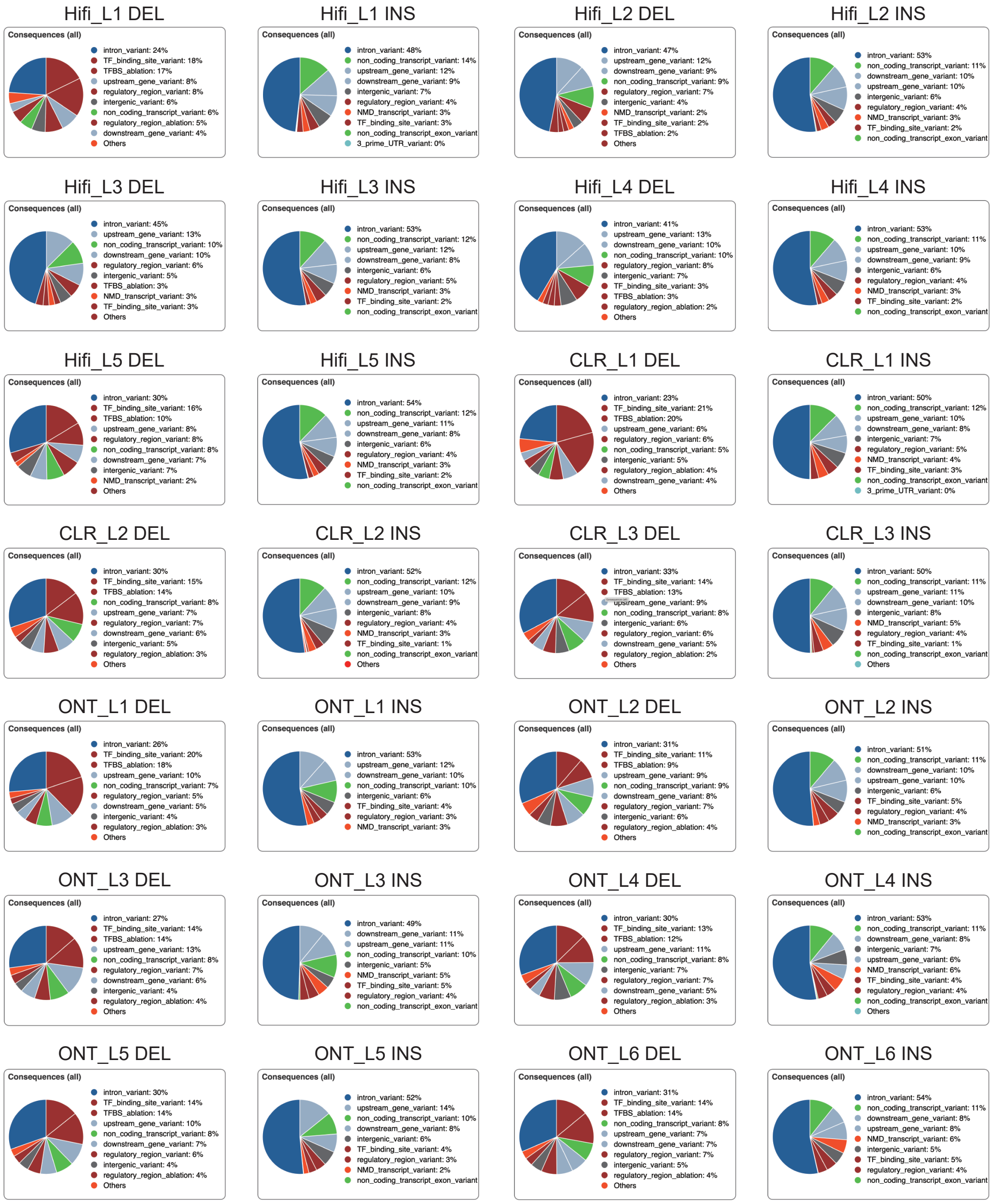

Supplementary Figure 1: The consequences annotation of unique TP SVs with correct GT detected by VolcanoSV for all 14 libraries using Ensembl Variant Effect Predictor (VEP). "Unique TPs with correct GT" refer to additional true positive (TP) SVs with correct genotypes (GT) detected by VolcanoSV compared to the second-ranked tool in each dataset. Source data are provided as a Source Data file.

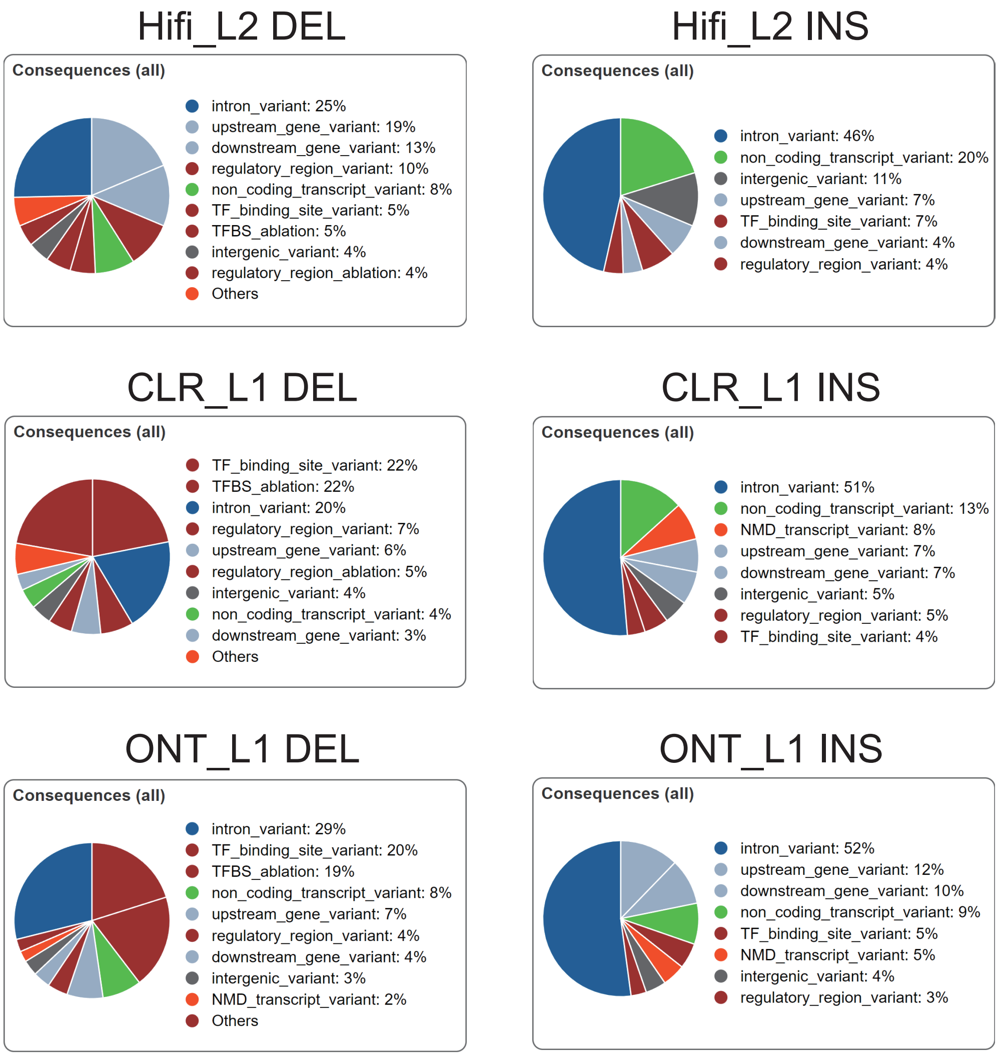

Supplementary Figure 2: The consequences annotation of unique TP SVs detected by VolcanoSV for three representative libraries using Ensembl Variant Effect Predictor (VEP). "Unique TPs" refer to unique true positive (TP) SVs detected by VolcanoSV compared to PAV, SVIM-asm, and Dipcall in each dataset. Source data are provided as a Source Data file.

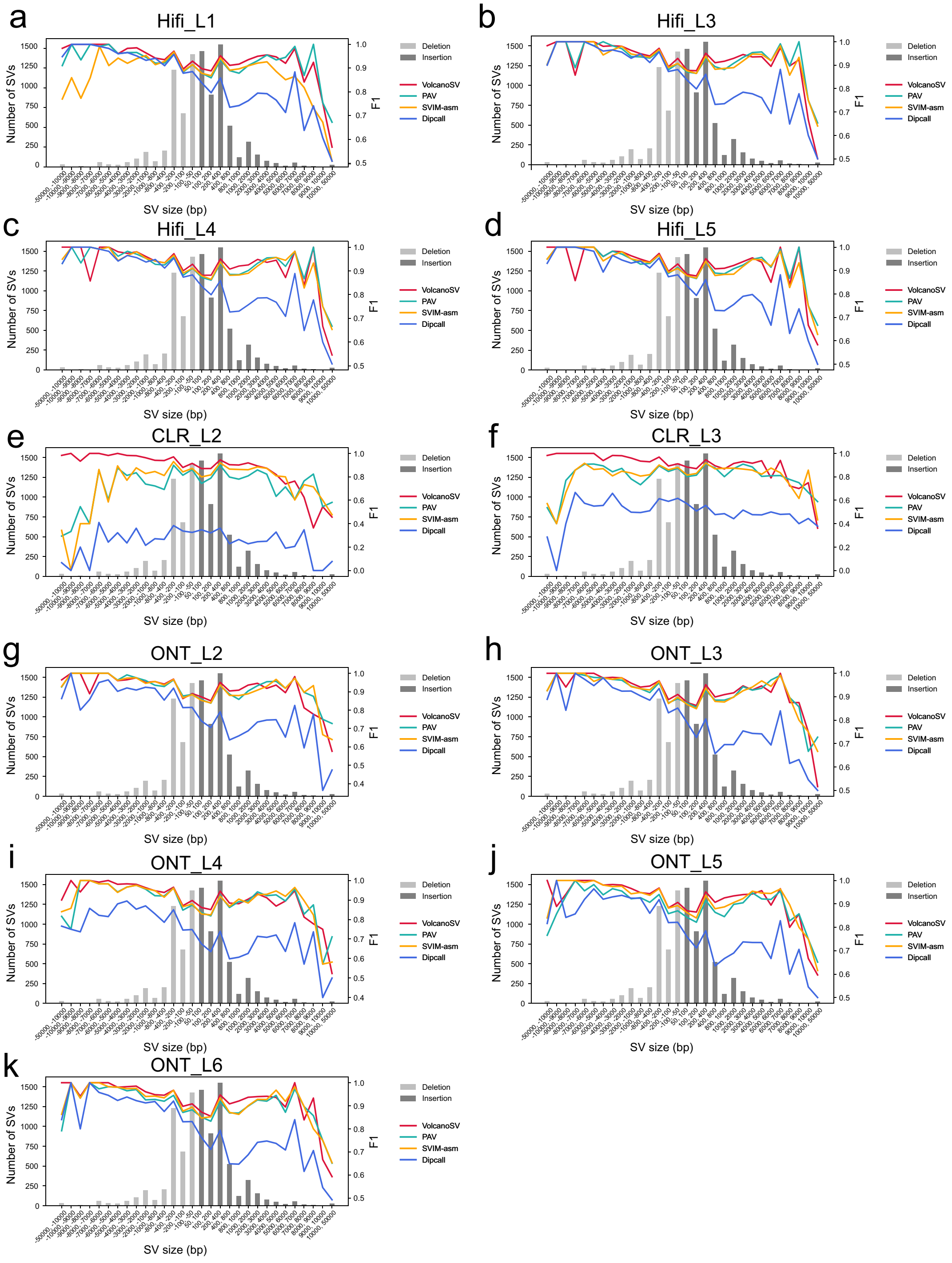

Supplementary Figure 3: **F1 accuracy of SV detection at different size ranges. (a-d)** F1 accuracy of SV detection on Hifi_L1, Hifi_L3, Hifi_L4, and Hifi_L5. **(e-f)** F1 accuracy of SV detection on CLR_L2 and CLR_L3. **(g-k)** F1 accuracy of SV detection on ONT_L2, ONT_L3, ONT_L4, ONT_L5, and ONT_L6. The negative size range represents deletions and the positive size range represents insertions. The bar plot shows benchmark SV distribution at different size ranges. The line plot shows the F1 score of four different methods. Source data are provided as a Source Data file.

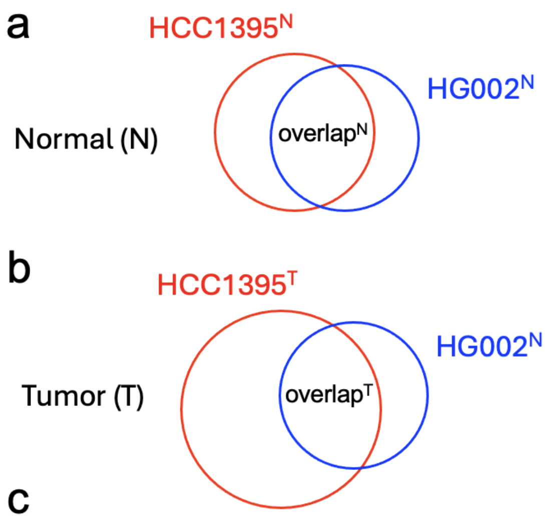

c

Equation 1: $overlap^N / overlap^T \approx 1$

Equation 2: $overlap^N / HCC1395^N - overlap^T / HCC1395^T > 0$

Equation 3: $overlap^N / HG002^N - overlap^T / HG002^N \approx 0$

d

TRA	VolcanoSV	pbsv
Recall	0.32	0.55
Equation 1	0.71	1.01
Equation 2	2.69%	14.41%
Equation 3	-4.87%	0.21%

INV	VolcanoSV	pbsv
Recall	0.29	0.48
Equation 1	1.23	1.31
Equation 2	9.73%	14.14%
Equation 3	5.37%	7.10%

DUP	VolcanoSV	pbsv
Recall	0.13	0.43
Equation 1	1.16	1.14
Equation 2	-0.16%	1.36%
Equation 3	5.75%	2.51%

Supplementary Figure 4: **Analyzing the low recall of complex SVs in real cancer datasets.** **(a)** $HCC1395^N$ denotes the number of SV calls by VolcanoSV for the HCC1395 normal library, $HG002^N$ for the HG002 library, and $overlap^N$ for the overlapping SV calls between these two call sets. **(b)** $HCC1395^T$ denotes the SV calls by VolcanoSV for the HCC1395 tumor library, $HG002^N$ for the HG002 library, $overlap^T$ for the overlapping SV calls between two sets. **(c)** Three equations to illustrate good metrics for SV callers. **(d)** The metric values for translocation (TRA), inversion (INV), and duplication (DUP) by VolcanoSV and pbsv. Source data are provided as a Source Data file.

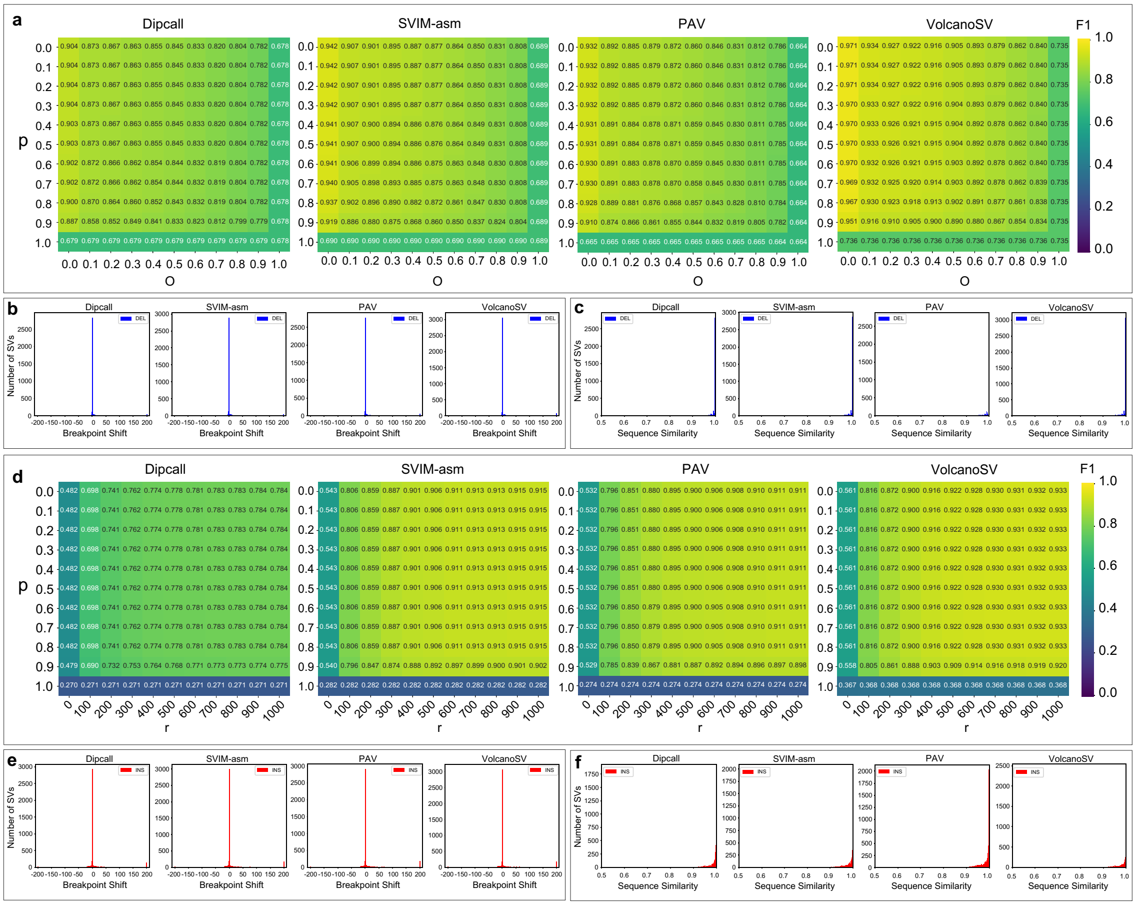

Supplementary Figure 5: **F1 accuracy by tuning different evaluation parameters and distribution of breakpoint shift and alternate allele sequence similarity for SVs on CLR_L1**. **(a)** Grid search F1 heatmap for deletions by different assembly-based tools. **(b)** Distribution of breakpoint shift for deletions by assembly-based tools. **(c)** Distribution of alternate sequence similarity for deletions by assembly-based tools. **(d-f)** Equivalent visual representations as shown in (a-c) for insertions. Source data are provided as a Source Data file.

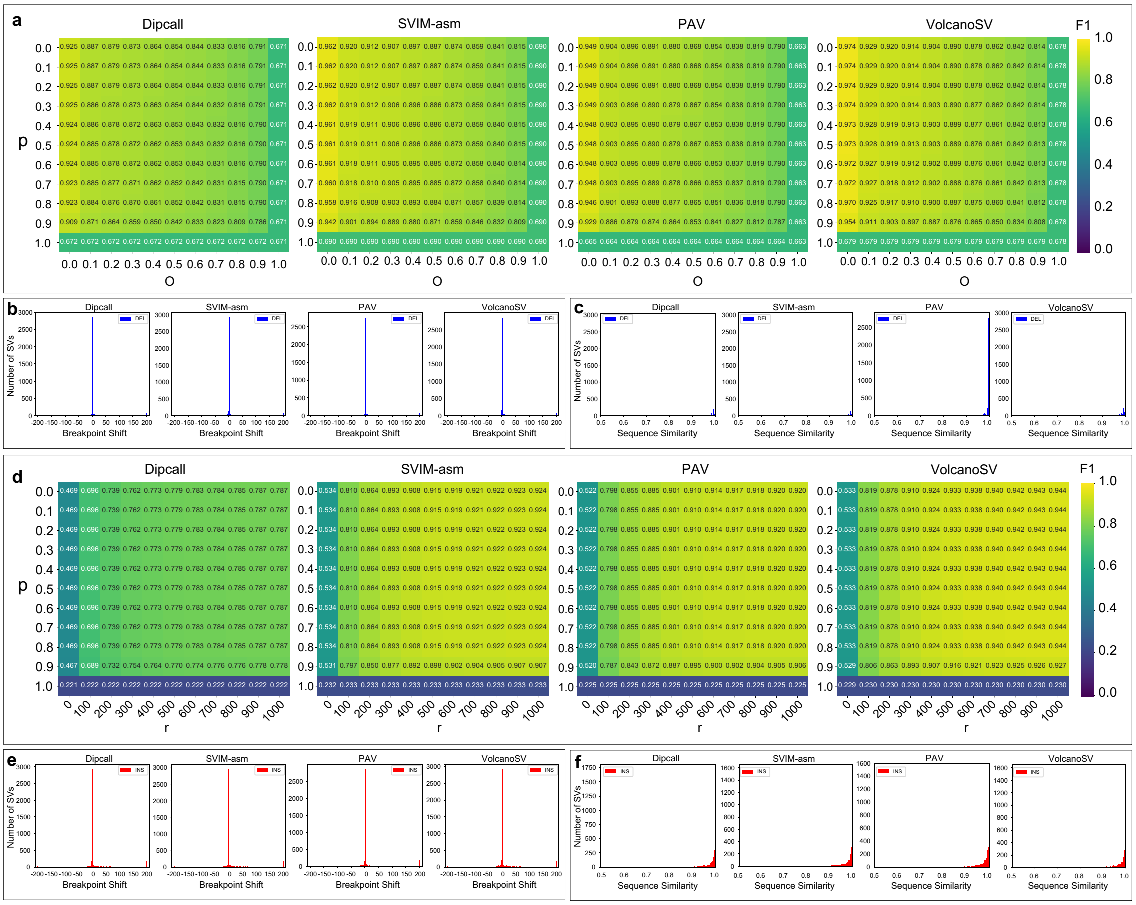

Supplementary Figure 6: **F1 accuracy by tuning different evaluation parameters and distribution of breakpoint shift and alternate allele sequence similarity for SVs on ONT_L1**. **(a)** Grid search F1 heatmap for deletions by different assembly-based tools. **(b)** Distribution of breakpoint shift for deletions by assembly-based tools. **(c)** Distribution of alternate sequence similarity for deletions by assembly-based tools. **(d-f)** Equivalent visual representations as shown in (a-c) for insertions. Source data are provided as a Source Data file.

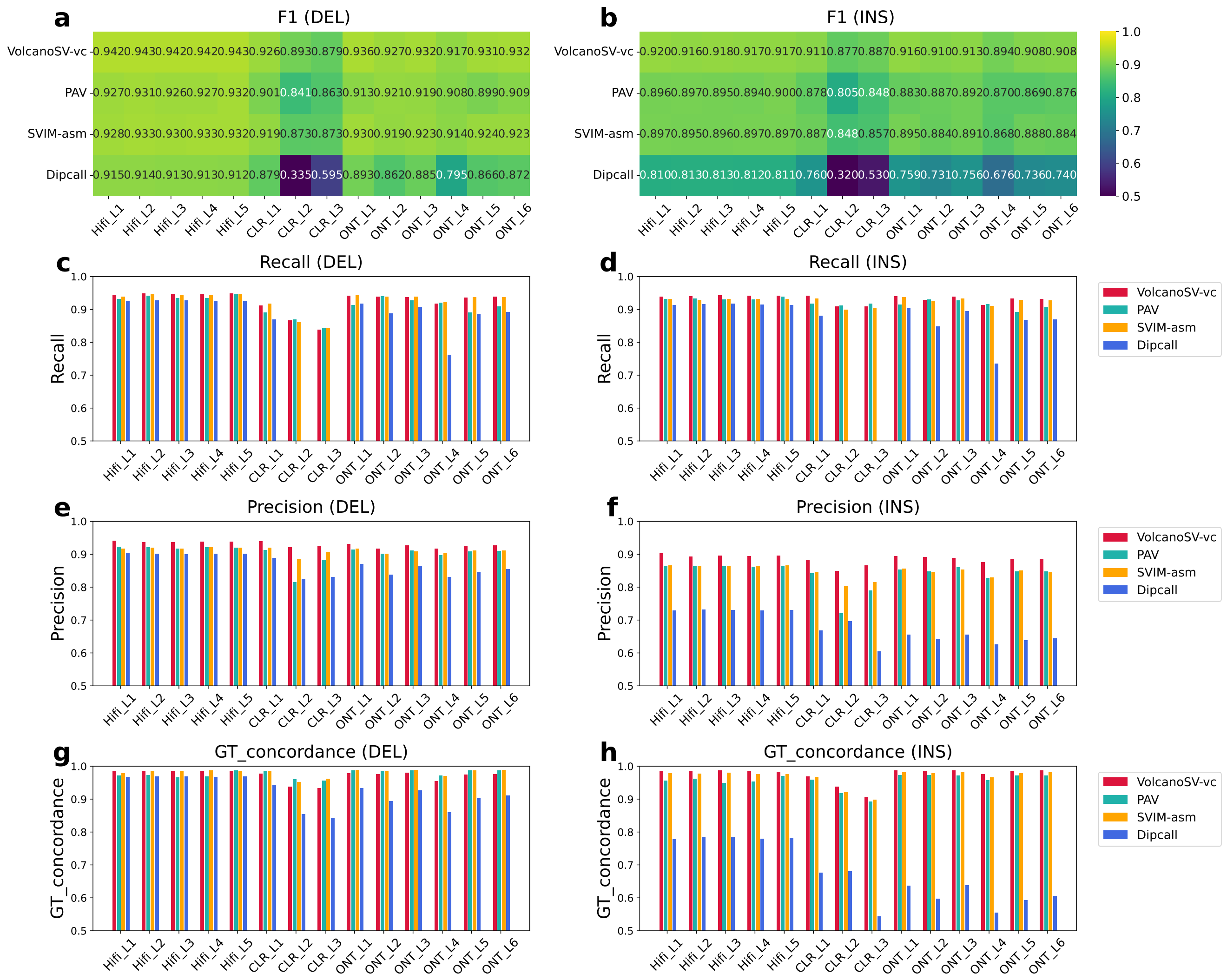

Supplementary Figure 7: **Cross datasets evaluation against GIAB HG002 benchmark. (a-b)** F1 heatmap for deletions (DEL) and insertions (INS) by four assembly-based tools. **(c-d)** Recall bar plots for deletions (DEL) and insertions (INS) by four assembly-based tools. **(e-f)** Precision bar plots for insertions deletion (DEL) and insertions (INS) by four assembly-based tools. **(g-h)** Genotype accuracy (represented by GT_concordance) bar plots for deletions (DEL) and insertions (INS) by four assembly-based tools: **VolcanoSV-vc**, PAV, SVIM-asm, and Dipcall. Source data are provided as a Source Data file.

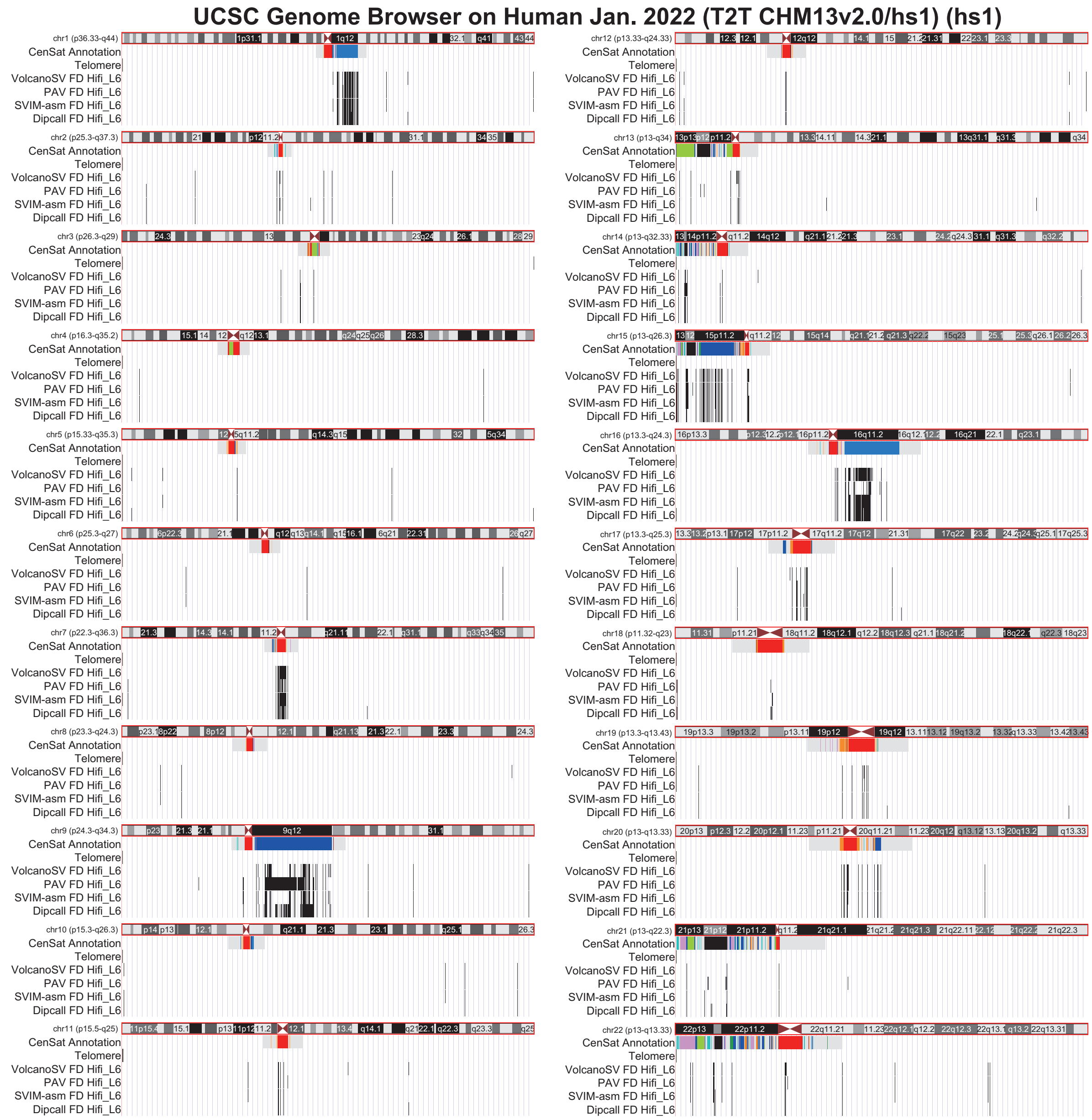

Supplementary Figure 8: **False discovery analysis in Hifi_L6 on the T2T-CHM13 reference.** The UCSC Genome Browser diplays six tracks for chromosomes 1 to 22: "CenSat Annotation" for centromere annotation, "Telomere" for telomere annotation, and individual tracks for false discoveries identified on T2T-CHM13 by VolcanoSV, PAV, SVIM-asm, and Dipcall in Hifi_L6, labeled as "VolcanoSV FD Hifi_L6, "PAV FD Hifi_L6", "SVIM-asm FD Hifi_L6", and "Dipcall FD Hifi_L6", respectively. Source data are provided as a Source Data file.

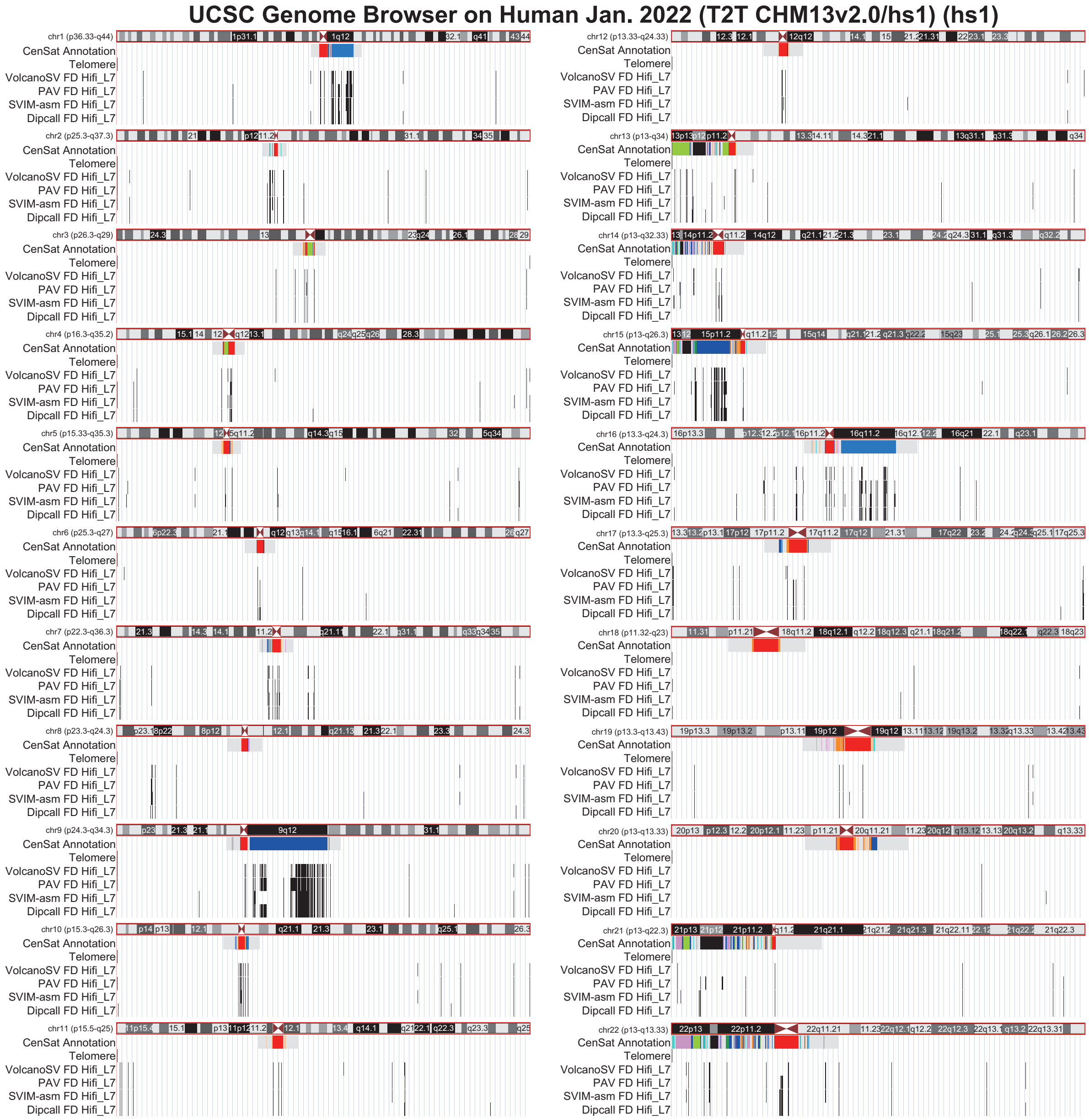

Supplementary Figure 9: **False discovery analysis in Hifi_L7 on the T2T-CHM13 reference.** The UCSC Genome Browser diplays six tracks for chromosomes 1 to 22: "CenSat Annotation" for centromere annotation, "Telomere" for telomere annotation, and individual tracks for false discoveries identified on T2T-CHM13 by VolcanoSV, PAV, SVIM-asm, and Dipcall in Hifi_L7, labeled as "VolcanoSV FD Hifi_L7, "PAV FD Hifi_L7", "SVIM-asm FD Hifi_L7", and "Dipcall FD Hifi_L7", respectively. Source data are provided as a Source Data file.

Supplementary References

[1] Talsania, K., Shen, T.-w., Chen, X., et al.: Structural variant analysis of a cancer reference cell line sample using multiple sequencing technologies. Genome Biology **23**(255) (2022)
