# Supplementary material for: VolcanoSV enables accurate and robust structural variant calling in diploid genomes from single-molecule long read sequencing
Source: Nat Commun. 2024 Aug 13;15:6956. doi: 10.1038/s41467-024-51282-0 (PMC11322167; doi:10.1038/s41467-024-51282-0)
Supplement: Supplementary file 3 — Reporting Summary [file 41467_2024_51282_MOESM3_ESM.pdf]

nature portfolio

Corresponding author(s): Xin Maizie Zhou

Last updated by author(s): Jul 14, 2024

Reporting Summary

Nature Portfolio wishes to improve the reproducibility of the work that we publish. This form provides structure for consistency and transparency in reporting. For further information on Nature Portfolio policies, see our Editorial Policies and the Editorial Policy Checklist.

Statistics

For all statistical analyses, confirm that the following items are present in the figure legend, table legend, main text, or Methods section.

n/a	Confirmed	
☐	☒	The exact sample size (*n*) for each experimental group/condition, given as a discrete number and unit of measurement
☐	☒	A statement on whether measurements were taken from distinct samples or whether the same sample was measured repeatedly
☒	☐	The statistical test(s) used AND whether they are one- or two-sided<br>*Only common tests should be described solely by name; describe more complex techniques in the Methods section.*
☒	☐	A description of all covariates tested
☒	☐	A description of any assumptions or corrections, such as tests of normality and adjustment for multiple comparisons
☒	☐	A full description of the statistical parameters including central tendency (e.g. means) or other basic estimates (e.g. regression coefficient) AND variation (e.g. standard deviation) or associated estimates of uncertainty (e.g. confidence intervals)
☐	☒	For null hypothesis testing, the test statistic (e.g. *F*, *t*, *r*) with confidence intervals, effect sizes, degrees of freedom and *P* value noted<br>*Give P values as exact values whenever suitable.*
☒	☐	For Bayesian analysis, information on the choice of priors and Markov chain Monte Carlo settings
☒	☐	For hierarchical and complex designs, identification of the appropriate level for tests and full reporting of outcomes
☐	☐	Estimates of effect sizes (e.g. Cohen's *d*, Pearson's *r*), indicating how they were calculated

Our web collection on statistics for biologists contains articles on many of the points above.

Software and code

Policy information about availability of computer code

Data collection	minimap2 2.24-r1122;<br>hifiasm 0.14 & 0.16<br>Hicanu 2.1.1;<br>Flye 2.9-b1768;<br>wtdbg2 2.5; Shasta 0.10.0; hapdup 0.5-iss10; purge_dups 1.2.5;<br>pbsim3 3.0.0;VISOR 1.1.2;<br>rasusa 0.6.0; samtools 1.18;<br>GRCh37 release 112; GRCh38 v.2.1.0; CHM13 v2.0
Data analysis	Longshot 0.4.1;<br>Dipcall;<br>SVIM-asm 1.0.2;<br>PAV freeze2;<br>Truvari 4.0.0; SURVIVOR 1.0.6; Flagger 0.3.3; VEP Ensembl;<br>VolcanoSV 1.0.0; SVIM 1.4.2; cuteSV 1.0.11; NanoVar 1.3.9; pbsv 2.6.2; DeBreak 1.0.2; Sniffles2 2.0.6<br>Nextdenovo 2.5.2<br>VolcanoSV v1.0

For manuscripts utilizing custom algorithms or software that are central to the research but not yet described in published literature, software must be made available to editors and reviewers. We strongly encourage code deposition in a community repository (e.g. GitHub). See the Nature Portfolio guidelines for submitting code & software for further information.

Data

Policy information about availability of data

All manuscripts must include a data availability statement. This statement should provide the following information, where applicable:

- Accession codes, unique identifiers, or web links for publicly available datasets
- A description of any restrictions on data availability
- For clinical datasets or third party data, please ensure that the statement adheres to our policy

PacBio CLR, Hifi, and ONT sequencing reads for HG002 are available at GIAB and NCBI. The high-confidence HCC1395 somatic SV callset and the Pacbio and ONT Tumor-Normal paired libraries of HCC1395 are publicly accessible at NCBI. PacBio Hifi sequencing reads for CHM13 are available at NCBI. Specific links for all 20 aforementioned real datasets are listed in Table 1. The Tier1 benchmark SV callset and high-confidence HG002 region were obtained from https://ftp-trace.ncbi.nlm.nih.gov/ReferenceSamples/giab/data/AshkenazimTrio/analysis/NIST_SVs_Integration_v0.6/. T2T assembly is publicly available at https://github.com/marbl/CHM13. The HG002 SD annotation file was downloaded from https://ftp-trace.ncbi.nlm.nih.gov/ReferenceSamples/giab/release/genome-stratifications/v3.4/. All files that support the findings of this study are available from https://doi.org/10.5281/zenodo.10456757 and https://doi.org/10.5281/zenodo.11641981. Data and code required to reproduce the results presented in this study are available at Source data.

Research involving human participants, their data, or biological material

Policy information about studies with human participants or human data. See also policy information about sex, gender (identity/presentation), and sexual orientation and race, ethnicity and racism.

Reporting on sex and gender	n/a
Reporting on race, ethnicity, or other socially relevant groupings	n/a
Population characteristics	n/a
Recruitment	n/a
Ethics oversight	n/a

Note that full information on the approval of the study protocol must also be provided in the manuscript.

Field-specific reporting

Please select the one below that is the best fit for your research. If you are not sure, read the appropriate sections before making your selection.

☒ Life sciences ☐ Behavioural & social sciences ☐ Ecological, evolutionary & environmental sciences

For a reference copy of the document with all sections, see nature.com/documents/nr-reporting-summary-flat.pdf

Life sciences study design

All studies must disclose on these points even when the disclosure is negative.

Sample size	We used the well-studied sample HG002 sequenced with 14 different long reads libraries, and tumor sample HCC1395 with four different normal and tumor libraries. We also used 9 simulated long reads datasets. This is a large sample size for whole genome analysis.
Data exclusions	No data was excluded from the analysis.
Replication	We have 14 different sequencing data from the same sample HG002. We also confirmed the reproducibility of the experimental findings.
Randomization	Our different long reads libraries originated from different research groups.
Blinding	We were unaware of the experimental conditions or phenotypic characteristics of the samples when analyzing the sequencing data. We were blinded to group allocation during data collection.

Reporting for specific materials, systems and methods

We require information from authors about some types of materials, experimental systems and methods used in many studies. Here, indicate whether each material, system or method listed is relevant to your study. If you are not sure if a list item applies to your research, read the appropriate section before selecting a response.

Materials & experimental systems

n/a	Involved in the study
☒	☐ Antibodies
☒	☐ Eukaryotic cell lines
☒	☐ Palaeontology and archaeology
☒	☐ Animals and other organisms
☒	☐ Clinical data
☒	☐ Dual use research of concern
☒	☐ Plants

Methods

n/a	Involved in the study
☒	☐ ChIP-seq
☒	☐ Flow cytometry
☒	☐ MRI-based neuroimaging

Plants

Seed stocks	n/a
Novel plant genotypes	n/a
Authentication	n/a
