# Supplementary material for: VolcanoSV enables accurate and robust structural variant calling in diploid genomes from single-molecule long read sequencing
Source: Nat Commun. 2024 Aug 13;15:6956. doi: 10.1038/s41467-024-51282-0 (PMC11322167; doi:10.1038/s41467-024-51282-0)
Supplement: Supplementary file 4 — Source Data [file 41467_2024_51282_MOESM4_ESM.zip › Source Data/Source Data.docx]

Zenodo refers to “<https://zenodo.org/records/11641981>”

DOI: **10.5281/zenodo.11641981**

## ********************Figures

**Figure 3**

Figure3/collect_truvari_2d.py

Figure3/plot_cross_data.py

Figure3/plot.sh

Figure3/Readme

Shared_Data/Cross_data_truvari4_eval

**Figure 4**

Figure4/F1_accuracy/line_bar_plot_multiple_tools_v2.py

Figure4/F1_accuracy/line_bar_plot_v1.py

Figure4/F1_accuracy/plot_f1.py

Figure4/F1_accuracy/plot.sh

Shared_Data/Cross_data_truvari4_eval

Figure4/UpSetPlot/plot_UpSet.py

Figure4/UpSetPlot/Data

Figure4/UpSetPlot/plot.sh

Figure4/UpSetPlot/tool_list_Hifi_L2.csv

Figure4/UpSetPlot/Readme

Figure4/Heatmap/RealData/plot_real_data.py

Figure4/Heatmap/RealData/Data

Figure4/Heatmap/RealData/plot.sh

Figure4/Heatmap/RealData/eval_list.csv

Figure4/Heatmap/SimulatedData

Figure4/Heatmap/SimulatedData/plot_sim_heatmap.py

Figure4/Heatmap/SimulatedData/Data

Figure4/Heatmap/SimulatedData/plot.sh

**Figure 5**

Figure5/CLR_L1/Recall_Precision_F1_subsampling_VC.py

Figure5/Hifi_L1/Recall_Precision_F1_subsampling_VC.py

Figure5/ONT_L1/Recall_Precision_F1_subsampling_VC.py

**Figure 6 and Supplementary Figure 5-6**

Figure6_SupplementaryFigure5-6/Heatmap/submit_single_Truvari.sbatch

Figure6_SupplementaryFigure5-6/Heatmap/vcf_filter.py

Figure6_SupplementaryFigure5-6/Heatmap/Truvari_results_heatmap_VC.py

Figure6_SupplementaryFigure5-6/Heatmap/Hifi_L1/Truvari_VolcanoSV_grid_search.sh

Figure6_SupplementaryFigure5-6/Heatmap/Hifi_L1/Truvari_Dipcall_grid_search.sh

Figure6_SupplementaryFigure5-6/Heatmap/Hifi_L1/Truvari_PAV_grid_search.sh

Figure6_SupplementaryFigure5-6/Heatmap/Hifi_L1/Truvari_SVIM-asm_grid_search.sh

Figure6_SupplementaryFigure5-6/Heatmap/Hifi_L1/tools_truvari_list.config

Figure6_SupplementaryFigure5-6/Heatmap/ONT_L1/Truvari_VolcanoSV_grid_search.sh

Figure6_SupplementaryFigure5-6/Heatmap/ONT_L1/Truvari_Dipcall_grid_search.sh

Figure6_SupplementaryFigure5-6/Heatmap/ONT_L1/Truvari_PAV_grid_search.sh

Figure6_SupplementaryFigure5-6/Heatmap/ONT_L1/Truvari_SVIM-asm_grid_search.sh

Figure6_SupplementaryFigure5-6/Heatmap/ONT_L1/tools_truvari_list.config

Figure6_SupplementaryFigure5-6/Heatmap/CLR_L1/Truvari_VolcanoSV_grid_search.sh

Figure6_SupplementaryFigure5-6/Heatmap/CLR_L1/Truvari_Dipcall_grid_search.sh

Figure6_SupplementaryFigure5-6/Heatmap/CLR_L1/Truvari_PAV_grid_search.sh

Figure6_SupplementaryFigure5-6/Heatmap/CLR_L1/Truvari_SVIM-asm_grid_search.sh

Figure6_S5_S6/Heatmap/CLR_L1/tools_truvari_list.config

Figure6_SupplementaryFigure5-6/Heatmap/README

Figure6_SupplementaryFigure5-6/Breakpoint_shift/Breakpoint_shift_VC.py

Figure6_SupplementaryFigure5-6/Breakpoint_shift/README

Figure6_SupplementaryFigure5-6/Sequence_similarity/Sequence_similarity_distribution_VC.py

Figure6_SupplementaryFigure5-6/Sequence_similarity/README

**Supplementary Figure 1**

SupplementaryFigure1/README

**Supplementary Figure2**

SupplementaryFigure2/README

**Supplementary Figure 3**

SupplementaryFigure3/line_bar_plot_multiple_tools_v2.py

SupplementaryFigure3/line_bar_plot_v1.py

SupplementaryFigure3/plot_f1.py

SupplementaryFigure3/plot.sh

SupplementaryFigure3/truvari_list.config

**Supplementary Figure 4**

SupplementaryFigure4/Data

SupplementaryFigure4/collect_data.py

SupplementaryFigure4/get_overlap.py

SupplementaryFigure4/reformat_volcanosv_vcf.py

SupplementaryFigure4/compare_pbsv.sh

SupplementaryFigure4/compare_volcanosv.sh

SupplementaryFigure4/collect_data.sh

SupplementaryFigure4/get_overlap_one_pair.sh

SupplementaryFigure4/run_pipeline.sh

SupplementaryFigure4/Readme

**Supplementary Figure 7**

SupplementaryFigure7/collect_truvari_2d.py

SupplementaryFigure7/plot_cross_data.py

SupplementaryFigure7/plot.sh

SupplementaryFigure7/Readme

**Supplementary Figure 8-9**

SupplementaryFigure8-9/Data/

SupplementaryFigure8-9/run.sh

SupplementaryFigure8-9/vcf2bed.py

SupplementaryFigure8-9/README

## ********************Tables

**Table2, Supplementary Table 1-3 and Supplementary Table 9-11**

Table2_SupplementaryTable1-3_SupplementaryTable9-11/Readme

Shared_Data/Cross_data_truvari4_eval

**Table 3**

Table3/run.sh

Table3/README

Table3/chm13.draft_v1.1.hets_combined.20211013.sorted.bed

Table3/check_het_site_overlap.py

Zenodo/Table3

**Supplementary Table 4**

SupplementaryTable4/gnomAD_Match/vcf_filter.py

SupplementaryTable4/gnomAD_Match/run.sh

SupplementaryTable4/gnomAD_Match/tool_list_gt.config

SupplementaryTable4/gnomAD_Match/submit_Truvari_template.slurm

SupplementaryTable4/Phased_Gene/run.sh

SupplementaryTable4/VEP/README

SupplementaryTable4/bin/TP-base_vcf_simplify.py

SupplementaryTable4/bin/vcf2sift.py

SupplementaryTable4/bin/vcf2vep.py

SupplementaryTable4/bin/vcf2bed.py

SupplementaryTable4/bin/gnomAD_AFfilter.py

SupplementaryTable4/bin/separate_INS_DEL.py

SupplementaryTable4/bin/phased_gene_analysis.py

SupplementaryTable4/bin/phased_gene_tp-gt.py

SupplementaryTable4/README

**Supplementary Table 5**

SupplementaryTable5/Data/

SupplementaryTable5/gnomAD_Match

SupplementaryTable5/gnomAD_Match/run.sh

SupplementaryTable5/VEP

SupplementaryTable5/VEP/README

SupplementaryTable5/README

**Supplementary Table 6-8**

SupplementaryTable6-8/Readme

**Supplementary Table 12-14**

SupplementaryTable12-14/eval_indel_v2.py

SupplementaryTable12-14/eval_snp_include_bed.py

SupplementaryTable12-14/HG002_GRCh37_1_22_v4.2.1_benchmark_noinconsistent_reformatted.bed

Zenodo/INDEL

Zenodo/SNP
